# The Artificial-Feeding System with a Lactic Acid Bacteria-Fermented Diet, Compared with Parent Feeding, Is Associated with Tract-Wide Microbiota Shifts and Coordinated Developmental Indices in Squabs

**DOI:** 10.3390/ani16142145

**Published:** 2026-07-10

**Authors:** Qijun Liang, Jinquan Xi, Shihong Liu, Tieshan Xu, Xinli Zheng, Li Zhang, Shudai Lin, Lizhi Lu, Zongxi Cao, Asmaa Taha Yaseen Kishawy, Lihong Gu

**Affiliations:** 1 Institute of Animal Science & Veterinary Medicine, Hainan Academy of Agricultural Sciences, Haikou 571100, China; 2 Haikou Coconut Pigeon Delicacy Breeding Co., Ltd., Haikou 571100, China; 3 Tropical Crops Genetic Resources Institute, Chinese Academy of Tropical Agricultural Sciences, Haikou 571101, China; 4 College of Coastal Agricultural Sciences, Guangdong Ocean University, Zhanjiang 524088, China; 5 State Key Laboratory for Managing Biotic and Chemical Threats to the Quality and Safety of Agro-Products, Institute of Animal Husbandry and Veterinary Medicine, Zhejiang Academy of Agricultural Sciences, Hangzhou 310021, China; 6 Department of Nutrition and Clinical Nutrition, Faculty of Veterinary Medicine, Zagazig University, Zagazig 44500, Egypt

**Keywords:** gut microbiota, *Limosilactobacillus*, squab, artificial feeding, fermented diet

## Abstract

Early-life dietary transition is a critical window for gut microbiota assembly and intestinal maturation in squabs. This study compared the overall artificial-feeding system using a lactic acid bacteria-fermented diet (AF) with a parent-feeding system (PF) during 18–25 days of age. Because AF differed from PF in both diet exposure and feeding ecology, including manual feeding, absence of parental feeding and crop milk, altered feeding rhythm, and potential loss of parental microbial transmission, the objective was to characterize associations between the two systems rather than to isolate the effect of fermentation alone. Squabs were assigned to AF or PF for 7 days. Microbial communities in the duodenum, jejunum, ileum, and rectum were profiled by 16S rRNA sequencing, and body size traits, organ indices, jejunal morphology, muscle histology, serum biochemical indicators, and muscle composition were assessed. The AF system, characterized by lower pH, a higher acid value, and detectable lactic acid bacteria in the feed, was associated with higher alpha diversity in multiple segments and distinct beta-diversity profiles relative to PF. Across intestinal regions, lactobacilli-related genera were enriched, with *Limosilactobacillus* consistently identified as the most discriminant genus and, together with *Lactobacillus*, occupying central positions in exploratory co-occurrence networks. AF was also associated with a greater jejunal villus height and villus height-to-crypt depth ratio, larger muscle fiber dimensions, higher intramuscular fat content with increased levels of selected polyunsaturated fatty acids, altered lipid-related serum indicators, and higher organ indices, including the bursa of Fabricius. PICRUSt2 further suggested differences in predicted functional potential related to carbohydrate, amino acid, lipid, and cofactor/vitamin metabolism, although these outputs represent inference from 16S data rather than directly measured functions. Collectively, the AF system was associated with a lactobacilli-centered, tract-wide microbial signature and coordinated intestinal, muscular, and lipid-related phenotypes in squabs, but the findings should not be interpreted as evidence for fermentation alone.

## Simple Summary

Young pigeons (squabs) undergo a rapid dietary transition while the gut microbiota and intestine are still developing. In this study, we compared the overall artificial-feeding system using a lactic acid bacteria-fermented diet (AF) with a parent-feeding system (PF) from 18 to 25 days of age. Because the AF and PF systems differed simultaneously in diet type, feeding mode, parental contact, crop-milk exposure, feeding frequency, and potential vertical microbial transmission, our findings describe associations with the overall AF system rather than the isolated effect of fermentation alone. Using 16S rRNA sequencing of the duodenum, jejunum, ileum, and rectum, the AF system was associated with tract-wide shifts in microbial diversity and composition, including enrichment of lactobacilli-related genera, particularly *Limosilactobacillus*. *Limosilactobacillus* consistently emerged as the most discriminant taxon and together with *Lactobacillus* occupied central positions in exploratory co-occurrence networks. These microbiota features co-occurred with greater jejunal villus height, larger muscle fiber dimensions, higher intramuscular fat with increased levels of selected polyunsaturated fatty acids, altered lipid-related serum indicators, and higher organ indices, including the bursa of Fabricius. Overall, the AF system was associated with a lactobacilli-centered microbiota signature aligned with coordinated intestinal, muscular, and lipid-related developmental traits in squabs.

## 1. Introduction

The gut microbiota is increasingly regarded as a host “metabolic organ” that contributes to nutrient utilization, energy homeostasis, immune development, and overall health in poultry [1,2]. Growing evidence indicates that early-life gut microbial colonization and succession can exert long-lasting “programming” effects, shaping host physiology and influencing subsequent growth efficiency and health trajectories [3,4]. Because microbial assembly coincides with rapid intestinal maturation, changes in microbial composition and function during this period may be closely linked to epithelial architecture, metabolic outputs, and systemic biochemical profiles [5]. Thus, clarifying how early-life microbial communities respond to nutritional inputs is fundamental to understanding avian development.

Domestic pigeons (*Columba livia domestica*) are a typical altricial species with a distinctive early-life feeding pattern. Squabs rely entirely on parental crop milk during the first week after hatching and then gradually transition to a mixture of crop milk and regurgitated, partially digested solid feed provided by parent pigeons from approximately 7 to 25 days of age [6,7]. This transition coincides with an immature digestive system and a developing microbiota, which may predispose squabs to digestive disturbance and increased susceptibility to enteric challenge [8]. In addition, parental transmission strongly shapes early microbial exposure, potentially constraining microbial diversity and resilience during this critical window [9]. Although pigeon crop milk composition and early intestinal immune-microbial development have been described [6,7,8], simultaneous characterization of segment-specific microbiota responses along the duodenum, jejunum, ileum, and rectum during this transition period remains limited in young pigeons.

Dietary manipulation is an effective strategy to reshape gut microbial ecology [10]. Fermented feeds, which can provide viable lactic acid bacteria, organic acids, and fermentation-derived bioactive metabolites, have been reported to promote beneficial colonization, suppress opportunistic taxa, and support gastrointestinal function in monogastric animals, including pigs and chickens [11,12]. Fermentation typically lowers pH and increases organic acid concentrations, thereby altering luminal conditions and ecological selection pressures that govern microbial assembly [13]. However, in squabs, an artificial-feeding system with fermented feed also differs from parent feeding in feeding behavior, parental care, crop-milk intake, and vertical microbial exposure. It therefore remains unclear whether the overall AF system is associated with a reproducible segment-resolved microbial signature across the intestinal tract and how such a system-level shift relates to developmental readouts during the feeding-transition period.

Recent taxonomic revisions have subdivided the former *Lactobacillus* genus into multiple genera, including *Limosilactobacillus* [14]. Both *Lactobacillus* and related lactobacilli are frequently linked to carbohydrate metabolism, organic acid production, and host epithelial and immune processes [15,16]. During early-life dietary transition, these taxa may respond preferentially to fermented substrates and may occupy central positions within microbial interaction networks, thereby contributing to community organization [17,18]. Nevertheless, evidence directly linking lactobacilli-related taxa to tract-wide microbial shifts and coordinated host phenotypes in squabs remains limited.

Accordingly, we compared the overall artificial-feeding system using a lactic acid bacteria-fermented diet (AF) with a parent-feeding system (PF) from 18 to 25 days of age to evaluate tract-wide, segment-resolved differences in the gut microbiota of squabs and their co-variation with selected developmental phenotypes. Microbial communities in the duodenum, jejunum, ileum, and rectum were profiled by 16S rRNA sequencing, and discriminant taxa and community features were evaluated using LEfSe and exploratory co-occurrence network analysis. Predicted functional profiles were inferred using PICRUSt2, and associations between microbial signatures and host phenotypes were assessed alongside measurements of body size traits, organ indices, jejunal morphology, muscle histology, serum biochemical indicators, and muscle composition. Because AF differed from PF in both diet type and feeding mode, the comparison addresses the AF system relative to PF rather than the isolated effect of fermentation alone.

## 2. Materials and Methods

### 2.1. Basal Diet and Fermented Feed

The basal diet was purchased from Dongguan Jinqian Feed Co., Ltd. (Dongguan, China), and its ingredient composition and nutrient levels are presented in Table 1. The fermented diet was produced by fermenting the basal diet with a commercial composite microbial starter (Hainan Hongyi Modern Agriculture Co., Ltd., Haikou, China). The starter comprised *Lactobacillus plantarum*, *Lactobacillus reuteri*, and *Pediococcus acidilactici*, with viable counts of 1.00 × 10^8^, 1.00 × 10^8^, and 2.30 × 10^8^ CFU/g, respectively. Briefly, 2 kg of starter was inoculated into 1000 kg of moistened basal diet, corresponding to a starter inclusion rate of 0.2% (*w*/*w*), and sterile water was added to achieve a target moisture content of approximately 40%. The mixture was homogenized, packed into plastic bags fitted with one-way valves, and incubated at 30 °C for 7 days. The analyzed nutrient composition of the diet before and after fermentation is reported in Table 2 (as-fed basis), with dry-matter-basis values provided for comparison. The fermented diet contained 7 × 10^5^ CFU/g lactic acid bacteria after fermentation (Table 2). Based on the daily intake of 65–70 g feed/squab in AF, the estimated intake of lactic acid bacteria was approximately 4.55 × 10^7^ to 4.90 × 10^7^ CFU/squab/day. Organic acids (e.g., lactic acid and acetic acid) and substrate characteristics such as total sugars, dietary fiber, total phenolics, and total flavonoids were not measured in this study and are acknowledged as limitations.

### 2.2. Animals and Experimental Design

The experiment was conducted at the Xinhaifu Meat Pigeon Breeding Base (Haikou, China). All procedures involving animals were reviewed and approved by the Animal Ethics Committee of Hainan Academy of Agricultural Sciences (Approval No. HNXMSY-20240606). Forty breeding pairs with comparable body weight and good health were selected and managed under artificial incubation combined with a 2 + 3 rearing model (each breeding pair raised three squabs). At 18 days of age, 120 squabs were randomly assigned to two treatments: artificial feeding with fermented feed (AF) or continued parent feeding (PF). Each treatment comprised six replicates (experimental units), with 10 squabs per replicate. In the AF group, squabs were manually fed the fermented diet twice daily (06:00 and 18:00) at 65–70 g per squab per day. Breeding pigeons in both treatments received the same basal diet (Table 1) ad libitum. In the PF group, squabs remained with their parents and were fed crop milk and regurgitated, partially digested feed derived from the basal diet. Water was provided ad libitum throughout the trial. The loft was disinfected routinely, and vaccinations were administered according to the farm immunization program. The intervention lasted 7 days (18–25 days of age), and no mortality occurred during the experiment.

### 2.3. Body Measurements

At the end of the 7-day intervention (25 days of age), squabs were fasted for 12 h and weighed. The following morphometric traits were recorded: body diagonal length (distance from the shoulder joint to the ischial tuberosity), chest width (distance between the shoulder joints), chest depth (distance from the first thoracic vertebra to the anterior edge of the keel), keel length (distance from the tip of the keel cartilage to the distal end of the keel), shank circumference (circumference at the midpoint of the tibia), shank length (straight-line distance from the proximal shank joint to the third and fourth toes), and pelvis width (distance between the iliac crests).

### 2.4. Sample Collection

At the end of the 7-day intervention (25 days of age), squabs were fasted for 12 h and then humanely euthanized by cervical dislocation performed by trained personnel. Exsanguination was carried out only after death to enable slaughter-performance measurements. For body size and slaughter-related traits, individual birds within each replicate were measured and replicate means were used for statistical analysis. For microbiota and serum biochemical analysis, equal aliquots from birds within the same replicate were pooled to obtain one composite sample per replicate (n = 6 per treatment). For histology and muscle composition, one representative bird per replicate, selected from birds close to the replicate mean body weight, was used. Blood was collected from the jugular vein; serum was separated by centrifugation (3000× *g*, 8 min, 4 °C) and stored at −80 °C for biochemical analyses. Samples from the left pectoralis major (breast muscle) were collected for pH, drip loss, color, and shear force measurements. An additional portion of left breast muscle was stored at −20 °C for proximate composition, amino acid, and fatty acid analyses. For histological evaluation of myofiber diameter and cross-sectional area, approximately 5 mm^3^ pieces of breast and leg muscle were fixed in 4% paraformaldehyde.

Before dissection, body weight was recorded. The abdominal cavity was opened, and the heart, liver, spleen, bursa of Fabricius, pancreas, and lungs were excised and weighed immediately. Organ indices were calculated as organ weight/body weight (g/kg body weight). Intestinal contents from the duodenum, jejunum, ileum, and rectum, defined here as the terminal intestinal segment immediately proximal to the cloaca [19,20], were collected aseptically and stored at −80 °C for microbiota analysis. In addition, a 1 cm jejunal segment was rinsed with saline to remove luminal contents and fixed in 4% paraformaldehyde for morphological assessment.

### 2.5. Euthanasia, Slaughter Performance and Meat Quality

After euthanasia and subsequent exsanguination, feathers were removed and carcass weight was recorded. Semi-eviscerated weight, full-eviscerated weight, breast muscle weight, thigh muscle weight, and abdominal fat weight were then measured. Dressing percentage was calculated as carcass weight divided by pre-slaughter body weight. Semi-eviscerated and full-eviscerated yields were calculated as semi-eviscerated or full-eviscerated weight, respectively, divided by pre-slaughter body weight. Breast and thigh muscle yields were calculated as breast or thigh muscle weight divided by full-eviscerated weight. Abdominal fat percentage was calculated as abdominal fat weight divided by the sum of full-eviscerated weight and abdominal fat weight.

At 45 min postmortem, breast muscle pH was measured by inserting a pH electrode (Testo, Baden-Württemberg, Germany) into three sites, and the mean value was recorded. Breast muscle samples were then stored at 4 °C for 24 h, after which pH was measured again. At the same postmortem time point, color parameters, including lightness (L*), redness (a*), and yellowness (b*), were measured in triplicate at different locations using a portable colorimeter (Konica Minolta, Tokyo, Japan). Drip loss was determined by placing breast muscle samples in plastic bags at 4 °C for 24 h; samples were then blotted dry with filter paper, reweighed, and drip loss was expressed as the percentage weight loss relative to the initial weight. Shear force was measured using a digital meat tenderness meter (C-LM3B; Harbin, China).

### 2.6. Morphological Analysis

Breast muscle, leg muscle, and jejunal tissues fixed in 4% paraformaldehyde were processed using standard histological procedures, including dehydration, trimming, paraffin embedding, sectioning, hematoxylin and eosin (H&E) staining, and coverslipping. Whole-slide images were acquired using a panoramic slide scanner (3DHISTECH, Budapest, Hungary). Digital images were analyzed in Image-Pro Plus 6.0 to quantify muscle fiber diameter and cross-sectional area, as well as jejunal villus height and crypt depth; the villus height-to-crypt depth ratio (VH/CD) was calculated. To ensure comparability, images for each tissue type were captured using identical scanning settings, and scale bars were applied during analysis. Representative images were selected from sections with intact orientation and minimal processing artifacts; however, some villus disruption may arise during fixation, embedding, or sectioning and is acknowledged as a limitation.

### 2.7. Analysis of Nutritional Components in Muscle

Approximately 200 mg of freeze-dried muscle was used for amino acid profiling following the method of Heinrikson and Meredith [21]. Samples were hydrolyzed in 6 mol/L hydrochloric acid at 110 °C for 24 h. Amino acids, except tryptophan, methionine, and cysteine, were quantified using an amino acid analyzer (Hitachi L-8900, Tokyo, Japan). Methionine and cysteine were subjected to performic acid oxidation prior to hydrolysis in 7.5 mol/L hydrochloric acid at 110 °C for 24 h. Tryptophan was determined after alkaline hydrolysis with lithium hydroxide (LiOH) at 110 °C for 22 h and quantified by high-performance liquid chromatography (Agilent 1200 Series, Santa Clara, CA, USA).

Muscle fatty acid composition was analyzed using a targeted metabolomics workflow. Briefly, 50 mg of muscle was extracted with 1 mL dichloromethane:methanol (1:1, *v*/*v*) containing mixed internal standards in a 2 mL centrifuge tube and homogenized. After centrifugation (13,000× *g*, 10 min), the supernatant was collected and evaporated to dryness under nitrogen. The residue was reconstituted in 0.5 mol/L sodium hydroxide in methanol, and a 100 µL aliquot was transferred to a vial for analysis. Fatty acids were analyzed by gas chromatography–mass spectrometry (GC–MS) using an Agilent 8890B gas chromatograph coupled to an Agilent 5977B/7000D mass spectrometer (Agilent Technologies, Santa Clara, CA, USA) with electron impact (EI) ionization at 70 eV. Compounds were identified and quantified using MassHunter software (v10.0.707.0), and quality-control samples were included throughout the run to monitor system stability.

### 2.8. Detection of Serum Biochemical Indicators

Serum biochemical indicators were analyzed by the Beijing Huaying Biotechnology Research Institute. The measured variables included total protein (TP), alanine aminotransferase (ALT), triglyceride (TG), total cholesterol (TC), high-density lipoprotein cholesterol (HDL), low-density lipoprotein cholesterol (LDL), hormone-sensitive lipase (HSL), lipoprotein lipase (LPL), fatty acid synthase (FAS), leptin (LEP), adiponectin (ADP), and thyroid-stimulating hormone (TSH). Serum samples were thawed at 4 °C and mixed thoroughly. Biochemical parameters were measured using an automated biochemical analyzer (Mindray, Shenzhen, China) with the corresponding commercial reagent kits supplied by China National Pharmaceutical Group Corporation, North Control Co., Ltd. (Shijiazhuang, China).

### 2.9. 16S rRNA Gene Sequencing and Bioinformatic Analysis

Microbiota profiling of intestinal contents was performed by Shenzhen Huitong Biotechnology Co., Ltd. (Shenzhen, China). Total microbial DNA was extracted using the service provider’s standardized workflow for intestinal-content microbiome samples; DNA concentration and purity were assessed by NanoDrop, and DNA integrity was verified by 1.2% agarose gel electrophoresis. The V3–V4 hypervariable region of the bacterial 16S rRNA gene was amplified with primers 341F (5′-ACTCCTACGGGAGGCAGCA-3′) and 806R (5′-GGACTACHVGGGTWTCTAAT-3′). Each 25 μL PCR reaction contained 5 μL 5× reaction buffer, 5 μL 5× GC buffer, 2 μL dNTPs (2.5 mM each), 1 μL of each primer (10 μM), 2 μL template DNA, 8.75 μL ddH_2_O, and 0.25 μL Q5 DNA polymerase. Thermal cycling conditions were 98 °C for 2 min; 25–30 cycles of 98 °C for 15 s, 55 °C for 30 s, and 72 °C for 30 s; followed by 72 °C for 5 min. Amplicons were purified with Vazyme VAHTS DNA Clean Beads, quantified with Quant-iT PicoGreen on a BioTek FLx800 microplate reader, and used to construct libraries with the TruSeq Nano DNA LT Library Prep Kit (Illumina, San Diego, CA, USA). Libraries were quality-checked with an Agilent Bioanalyzer and sequenced on an Illumina MiSeq platform using paired-end 2 × 300 bp chemistry.

Raw paired-end reads were processed in QIIME2 (v2019.4) [22]. Primers and adapters were removed with Cutadapt (v2.8) [23], and denoising, paired-read merging, chimera removal, and ASV generation were performed with DADA2 (v1.16) [24]; singleton ASVs were removed. Across the 48 pooled samples, 3,311,262 raw reads were generated, corresponding to 55,220–90,873 reads/sample (mean 68,984.6). After quality filtering, denoising, merging, chimera removal, and singleton removal, 2,100,850 non-singleton reads remained, corresponding to 28,622–61,263 reads/sample (mean 43,767.7). Taxonomy was assigned with the q2-feature-classifier plugin [25] against the SILVA database (release 132) [26]. Alpha diversity (Shannon, Simpson, and Chao1) and Bray–Curtis beta diversity were calculated after rarefaction to 28,622 reads/sample. Principal coordinate analysis (PCoA) was used to visualize community structure, and PERMANOVA with 999 permutations was used to evaluate group separation within each intestinal segment. Relative abundance at the phylum and genus levels was calculated as the proportion of reads assigned to each taxon in the rarefied table for each sample. LEfSe was performed using the Huttenhower Lab Galaxy implementation [27] with α = 0.05 and an LDA score threshold of 4.0. Co-occurrence networks were generated as exploratory analyses from correlation matrices of dominant microbial features within each segment; taxa with mean relative abundance < 0.1% were excluded, and edges with |r| > 0.6 and nominal *p* < 0.05 were retained. Treatment-specific subnetwork summary metrics, including numbers of vertices and edges, density, clustering, transitivity, and modularity, were summarized when available from the service-provider output. Because correlation-based networks from compositional 16S data are sensitive to false positives, these networks were interpreted cautiously as descriptive rather than confirmatory. Functional potential was predicted with PICRUSt2 (v2.4.1) [28] and annotated against KEGG. PICRUSt2 outputs were treated as predicted functional profiles rather than directly measured functions; sample-level NSTI values were not retained in the archived service output available for this revision. A detailed summary of sequencing depth and read retention for all pooled samples is provided in Table S6, and rarefaction curves are shown in Figures S1–S4. Detailed network-construction parameters and summary topological metrics are presented in Table S8.

### 2.10. Statistical Analysis

Statistical analyses were conducted using SPSS 25.0 (IBM, Armonk, NY, USA). The replicate served as the experimental unit (n = 6 per treatment). For body size and slaughter-related traits, individual measurements within each replicate were averaged before analysis. For microbiota and serum biochemical indicators, one composite sample per replicate was analyzed; for histology and muscle composition, one representative bird per replicate was used. Normality was checked with the Kolmogorov–Smirnov test and homogeneity of variances with Levene’s test. When assumptions were satisfied, AF and PF were compared using an independent-samples *t* test; otherwise, the Mann–Whitney U test was used. For beta diversity, group separation within each intestinal segment was additionally evaluated by PERMANOVA with 999 permutations. Spearman correlation analysis was used to explore associations between differential genera and host phenotypes, and only associations with |r| > 0.6 and nominal *p* < 0.05 are displayed. Because a large number of pairwise tests were involved, correlation results were interpreted as exploratory and not as evidence of causality. Data are presented as mean ± SEM. Statistical significance was set at *p* < 0.05, and figure legends use * *p* < 0.05, ** *p* < 0.01, and *** *p* < 0.001.

## 3. Results

### 3.1. Changes in Nutrient Composition of Diets Before and After Fermentation

After fermentation, the diet exhibited higher moisture content and lower pH, accompanied by an increased acid value and detectable lactic acid bacteria (Table 2). On an as-fed basis, crude protein and crude ash concentrations were lower after fermentation, largely reflecting dilution by moisture; when expressed on a dry-matter basis, these differences were markedly attenuated (Table 2).

### 3.2. Effects of the AF System Versus PF on Body Size Traits in Squabs

**Figure 1:**
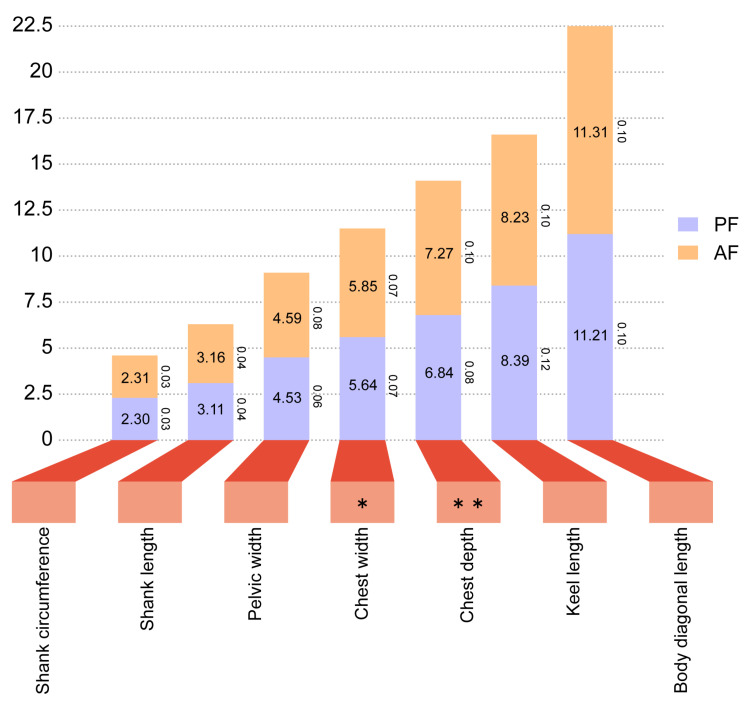
Effects of the AF system versus PF on body size traits in squabs (cm). Data are presented as mean ± SEM. * *p* < 0.05, ** *p* < 0.01. The tabulated data for Figure 1 are provided in Supplementary Table S1.

Body size traits are summarized in Figure 1. Compared with PF, AF was associated with greater chest width and chest depth (*p* < 0.05). No differences were observed between treatments for the remaining body size traits (*p* > 0.05).

### 3.3. Effects of the AF System Versus PF on Slaughter Performance and Meat Quality in Squabs

As shown in Table 3, semi-eviscerated yield was lower in AF than in PF (*p* < 0.05). This reduction occurred together with the higher organ indices observed for several visceral organs in AF (see Section 3.6), suggesting a possible shift in body-weight allocation toward internal organs rather than a generalized reduction in carcass development. Dressing percentage, full-eviscerated yield, breast muscle yield, thigh muscle yield, and abdominal fat percentage did not differ significantly between treatments (*p* > 0.05). As shown in Table 4, pH at 45 min postmortem was higher in AF than in PF (*p* < 0.05), whereas pH at 24 h, color, drip loss, and shear force were similar between treatments (*p* > 0.05).

### 3.4. Effects of the AF System Versus PF on Proximate Composition and Amino Acid Profile of Squab Muscle

**Figure 2:**
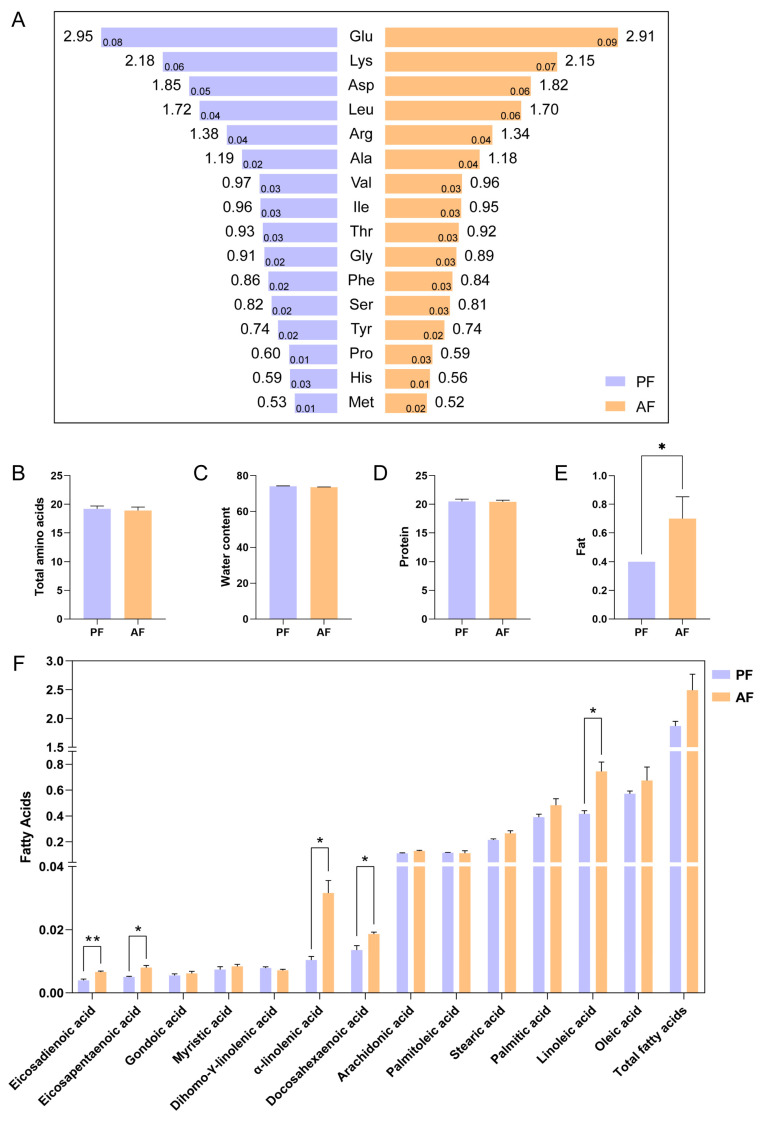
Effects of the AF system versus PF on muscle nutritional composition in squabs (g/100 g fresh weight). (**A**) Amino acid profile (g/100 g). (**B**–**E**) Proximate composition (g/100 g). (**F**) Fatty acid profile (g/100 g). Data are presented as mean ± SEM. * *p* < 0.05, ** *p* < 0.01. Abbreviations: EDA, eicosadienoic acid; EPA, eicosapentaenoic acid; ALA, α-linolenic acid; DHA, docosahexaenoic acid; LA, linoleic acid.

Intramuscular fat content was higher in AF than in PF (*p* < 0.05; Figure 2E), indicating greater lipid deposition in squab muscle in association with the AF system. In contrast, muscle moisture, crude protein, and amino acid composition did not differ between treatments (*p* > 0.05; Figure 2A–D).

### 3.5. Effects of the AF System Versus PF on Fatty Acid Composition of Squab Breast Muscle

Breast muscle fatty acid profiles are presented in Figure 2F. Relative to PF, AF increased eicosadienoic acid (EDA), eicosapentaenoic acid (EPA), α-linolenic acid (ALA), docosahexaenoic acid (DHA), and linoleic acid (LA) (*p* < 0.05), whereas the remaining fatty acids did not differ between treatments (*p* > 0.05).

### 3.6. Effects of the AF System Versus PF on Organ Development in Squabs

**Figure 3:**
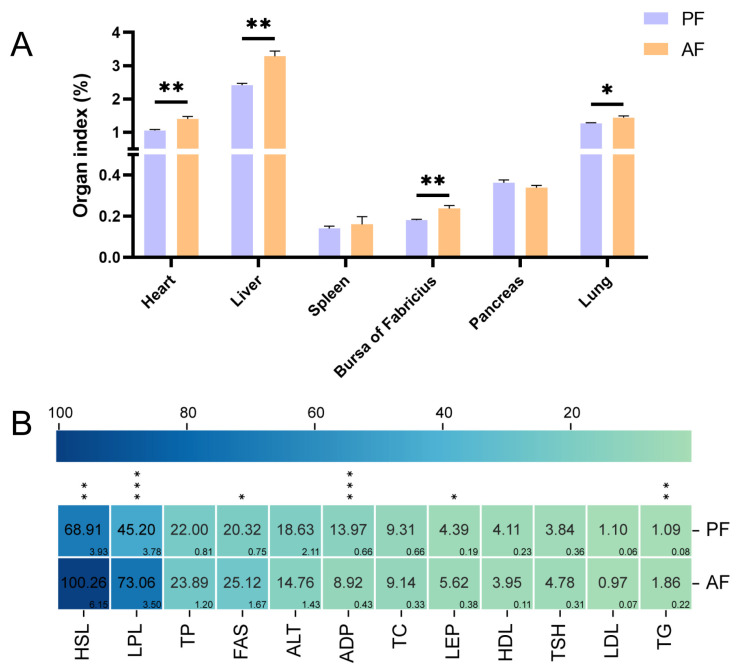
Effects of the AF system versus PF on organ indices and serum biochemical indicators in squabs. (**A**) Organ indices (g/kg body weight). (**B**) Serum biochemical indicators. Data are presented as mean ± SEM. * *p* < 0.05, ** *p* < 0.01, *** *p* < 0.001.

Organ indices (g/kg body weight) for the heart, liver, bursa of Fabricius, and lungs were higher in AF than in PF (*p* < 0.05; Figure 3A), whereas spleen and pancreas indices were similar between treatments (*p* > 0.05).

### 3.7. Effects of the AF System Versus PF on Serum Biochemical Indicators in Squabs

As shown in Figure 3B, AF increased serum hormone-sensitive lipase (HSL), lipoprotein lipase (LPL), fatty acid synthase (FAS), leptin (LEP), and triglyceride (TG) concentrations compared with PF (*p* < 0.05), whereas serum adiponectin (ADP) was lower (*p* < 0.05).

### 3.8. Effects of the AF System Versus PF on Muscle Fiber Characteristics and Jejunal Morphology

**Figure 4:**
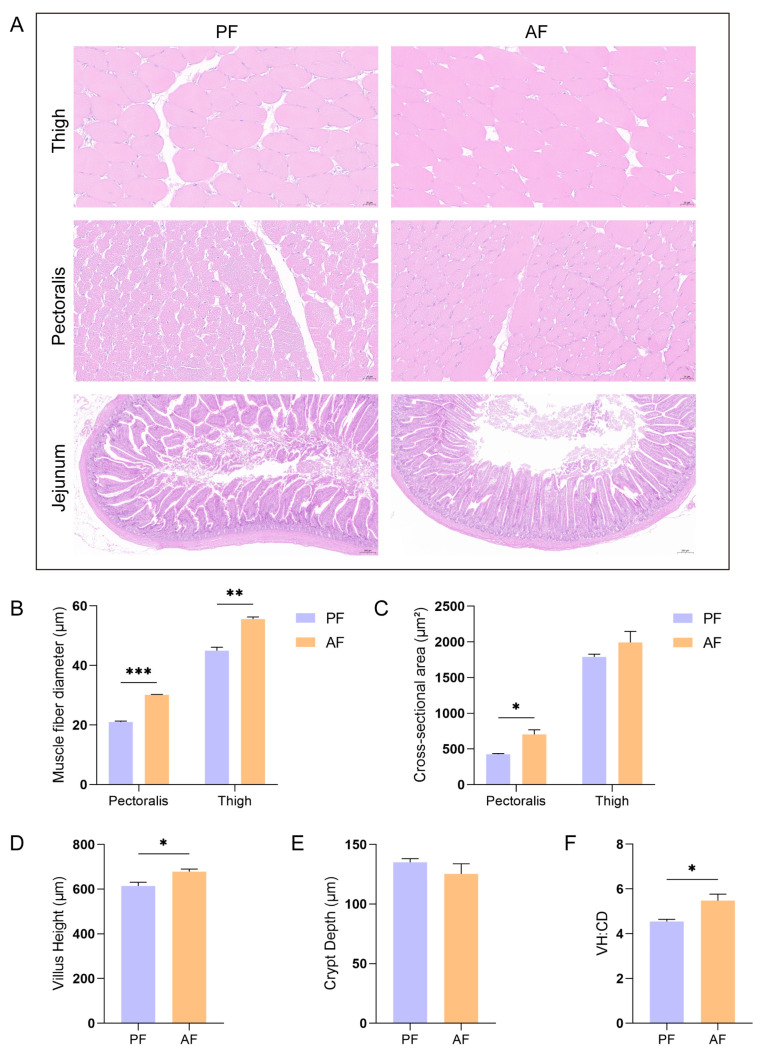
Effects of the AF system versus PF on muscle and jejunal morphology in squabs. (**A**) H&E-stained sections of pectoral muscle, thigh muscle, and jejunum (representative images captured at consistent magnification within each tissue type; scale bars shown). (**B**) Muscle fiber diameter (μm). (**C**) Muscle fiber cross-sectional area (μm^2^). (**D**) Jejunal villus height (μm). (**E**) Jejunal crypt depth (μm). (**F**) Villus height-to-crypt depth ratio (VH/CD). Data are presented as mean ± SEM. * *p* < 0.05, ** *p* < 0.01, *** *p* < 0.001.

Representative histological images are shown in Figure 4. Compared with PF, AF was associated with greater breast muscle fiber diameter and cross-sectional area (*p* < 0.05; Figure 4B,C). Leg muscle fiber diameter was also higher in AF than in PF (*p* < 0.05; Figure 4B). In the jejunum, AF increased villus height (*p* < 0.05; Figure 4D) and the villus height-to-crypt depth ratio (VH/CD) (*p* < 0.05; Figure 4F). The representative images should be interpreted together with the quantitative measurements because some local villus disruption may reflect processing artifact rather than treatment alone.

### 3.9. Effects of the AF System Versus PF on Intestinal Microbiota Alpha and Beta Diversity

**Figure 5:**
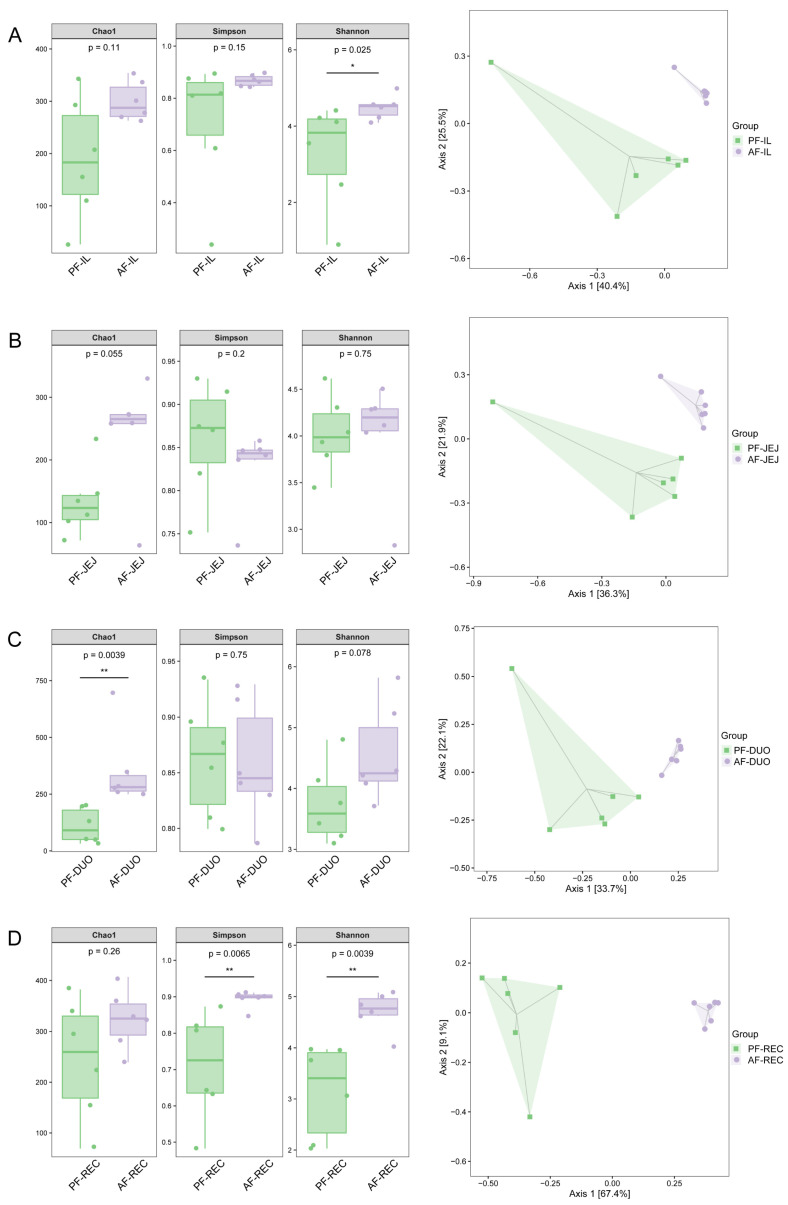
Effects of the AF system versus PF on alpha and beta diversity of the intestinal microbiota in squabs. Alpha diversity was assessed using the Shannon, Simpson, and Chao1 indices, and beta diversity was visualized by principal coordinate analysis (PCoA) based on Bray–Curtis dissimilarities. Group separation was evaluated by PERMANOVA with 999 permutations: ileum, *p* = 0.002; jejunum, *p* = 0.002; duodenum, *p* = 0.002; rectum, *p* = 0.002. (**A**) Ileum. (**B**) Jejunum. (**C**) Duodenum. (**D**) Rectum. Alpha diversity indices are presented as mean ± SEM. * *p* < 0.05, ** *p* < 0.01.

Alpha diversity was evaluated using the Shannon, Simpson, and Chao1 indices. Compared with PF, AF increased the Shannon index in the ileum (Figure 5A), the Chao1 index in the duodenum (Figure 5C), and the Simpson and Shannon indices in the rectum (Figure 5D). Principal coordinate analysis (PCoA) based on Bray–Curtis dissimilarities suggested separation between treatments across intestinal segments (Figure 5A–D). PERMANOVA confirmed significant group separation in all four segments (ileum, *p* = 0.002; jejunum, *p* = 0.002; duodenum, *p* = 0.002; rectum, *p* = 0.002), indicating that the AF system was associated with shifts in beta diversity. The complete PERMANOVA results for each intestinal segment are provided in Table S9.

### 3.10. Effects of the AF System Versus PF on Intestinal Microbiota Composition and Relative Abundance in Squabs

**Figure 6:**
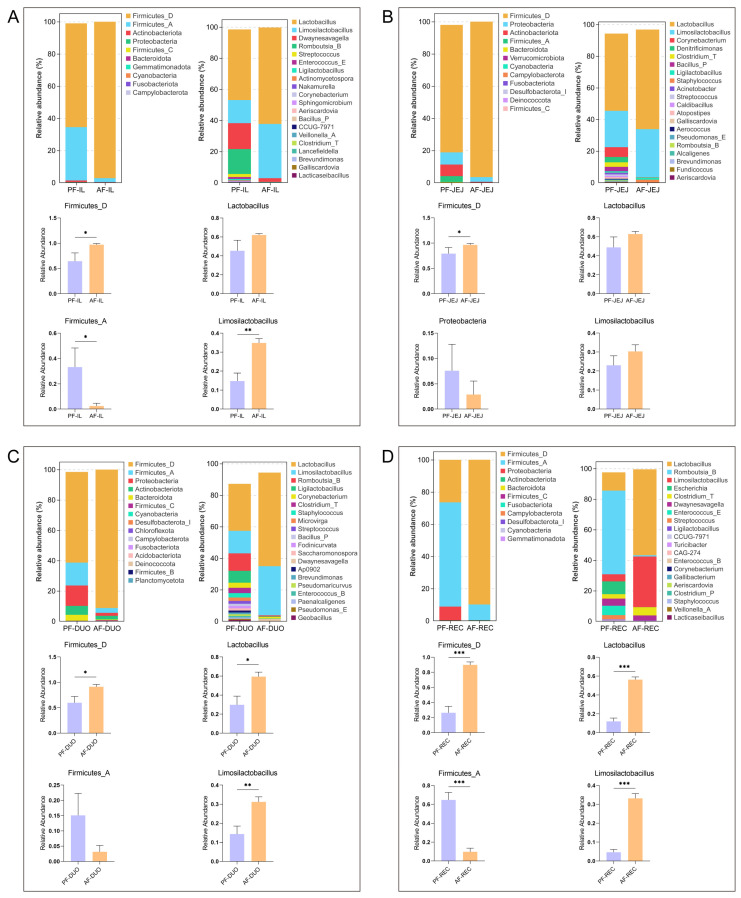
Effects of the AF system versus PF on intestinal microbiota composition and relative abundance in squabs. Relative abundance profiles at the phylum and genus levels are shown for the (**A**) ileum, (**B**) jejunum, (**C**) duodenum, and (**D**) rectum. Data are presented as mean ± SEM. * *p* < 0.05, ** *p* < 0.01, *** *p* < 0.001.

Figure 6 summarizes the relative abundance of microbial taxa at the phylum and genus levels across the duodenum, jejunum, ileum, and rectum. At the phylum level, AF increased the relative abundance of Firmicutes_D and decreased Firmicutes_A in the ileum (*p* < 0.05; Figure 6A). At the genus level, *Lactobacillus* and *Limosilactobacillus* were the dominant genera in the ileum, and *Limosilactobacillus* was more abundant in AF than in PF (*p* < 0.05; Figure 6A). In the jejunum, Firmicutes_D and Proteobacteria were the dominant phyla, whereas *Lactobacillus* and *Limosilactobacillus* remained among the dominant genera. In the duodenum and rectum, AF was likewise associated with enrichment of lactobacilli-related genera relative to PF. Relative abundance was calculated from the rarefied taxonomic table.

Overall, the AF system was associated with a segment-resolved shift characterized by redistribution between Firmicutes_D and Firmicutes_A and enrichment of lactobacilli-related genera, particularly *Lactobacillus* and *Limosilactobacillus*.

### 3.11. Identification of Marker Taxa by LEfSe and Exploratory Co-Occurrence Network Analysis

**Figure 7:**
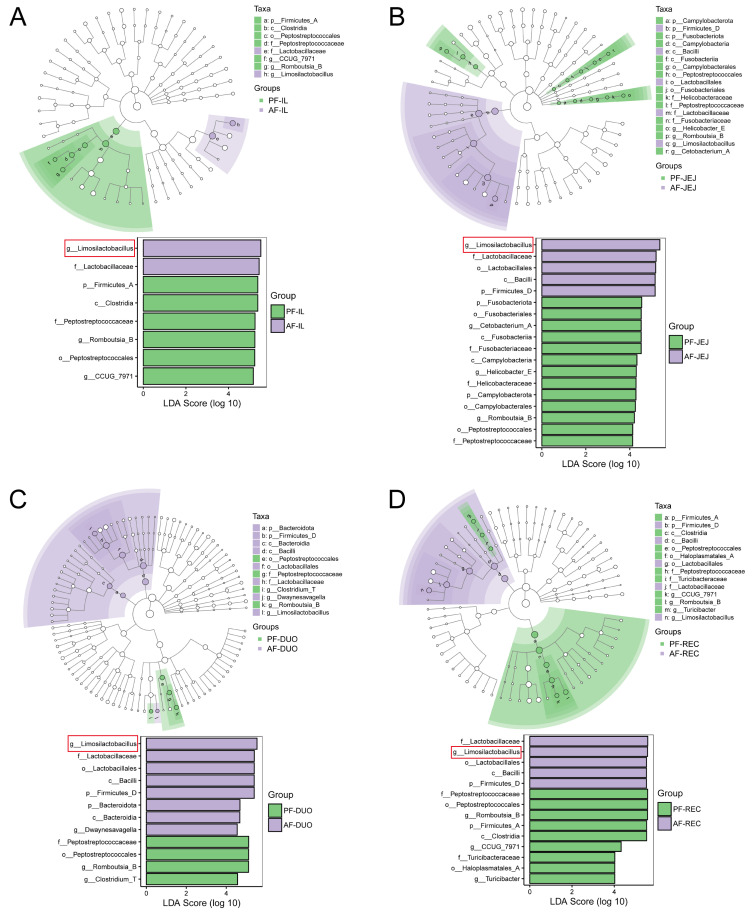
Differential taxa associated with the AF system versus PF identified by LEfSe. LEfSe results are shown for the (**A**) ileum, (**B**) jejunum, (**C**) duodenum, and (**D**) rectum.

**Figure 8:**
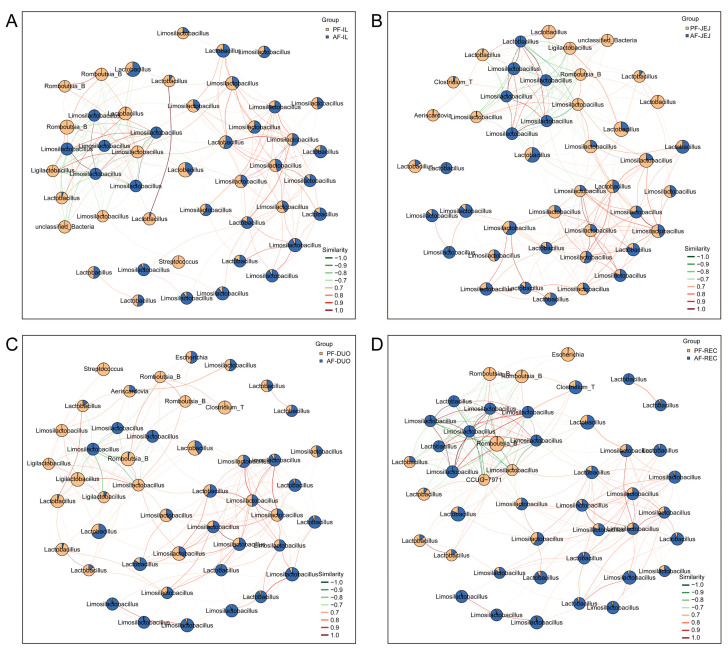
Exploratory co-occurrence network analysis of the intestinal microbiota in squabs, highlighting node characteristics of *Lactobacillus* and *Limosilactobacillus*. Networks are shown for the (**A**) ileum, (**B**) jejunum, (**C**) duodenum, and (**D**) rectum. Treatment-specific subnetwork topological metrics are summarized in Table S8, Part B.

LEfSe analysis identified taxa associated with the AF system relative to PF. As shown in Figure 7, *Limosilactobacillus* had the highest LDA scores across all four intestinal segments (ileum, 5.52; jejunum, 5.37; duodenum, 5.54; rectum, 5.58), indicating that it was the most discriminant genus between treatments under the present analytical settings. Exploratory co-occurrence network analysis further showed that *Lactobacillus* and *Limosilactobacillus* occupied central positions in all segments (Figure 8). Treatment-specific subnetwork metrics (Table S8, Part B) indicated that AF subnetworks had higher mean numbers of vertices and edges than PF subnetworks across all intestinal segments (duodenum: 164.667 vertices/1645.833 edges vs. 51.000/292.400; ileum: 184.167/1836.167 vs. 66.333/413.000; jejunum: 149.833/1528.167 vs. 65.600/421.800; rectum: 181.500/1841.500 vs. 54.000/323.667). In contrast, mean network density was lower in AF than in PF in all segments, whereas modularity differed only modestly and inconsistently among segments. Because one expected PF subnetwork file was absent from the service output for the duodenum and one for the jejunum, these two PF means were based on the available n = 5 subnetworks and should be interpreted cautiously. Overall, these topological summaries suggest larger but less dense AF-associated subnetworks. Because the networks were correlation based, they are interpreted here as descriptive topological patterns rather than evidence of direct ecological interaction or causality. The complete LEfSe results for each intestinal segment are provided in Table S7.

### 3.12. Lactobacillus and Limosilactobacillus as Key Taxa Associated with Microbiota Structure and Coordinated Host Phenotypes in Squabs

**Figure 9:**
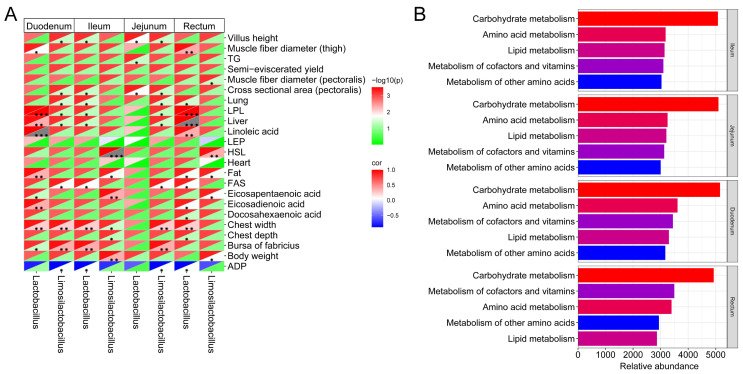
Associations between differential genera, host phenotypes, and predicted functions. (**A**) Spearman correlation heatmap between differential genera and phenotypic variables. (**B**) Predicted KEGG pathway enrichment generated by PICRUSt2. * *p* < 0.05, ** *p* < 0.01, *** *p* < 0.001.

Spearman correlation analysis was used to evaluate associations between differentially abundant genera and host phenotypic variables. As shown in Figure 9A, *Lactobacillus* abundance showed positive exploratory associations (|r| > 0.6, nominal *p* < 0.05) with multiple variables, including selected organ indices, body size traits, fatty acids, intramuscular fat content, lipid-related serum indicators, muscle fiber traits, and jejunal villus height. *Limosilactobacillus* abundance showed a similar co-variation pattern with several of these traits. Serum adiponectin (ADP) was negatively associated with both genera. Because a large number of pairwise comparisons were involved and no formal multiple-comparison correction was applied, these results should be interpreted as exploratory correlations rather than evidence of causality.

PICRUSt2-based prediction further suggested differences in inferred functional potential linked to lactobacilli-related taxa. As shown in Figure 9B, predicted KEGG ortholog enrichment involved pathways related to carbohydrate, amino acid, lipid, and cofactor/vitamin metabolism. These outputs represent predicted functional potential from 16S data rather than directly measured gene content, transcription, enzyme activity, or metabolite abundance and should therefore be interpreted cautiously.

## 4. Discussion

Early-life dietary transition represents a highly plastic period for intestinal microbiota assembly in squabs, during which shifts in substrate availability and luminal conditions can rapidly reshape community structure [3,4,29]. In the present study, the AF system differed from PF in both diet exposure and feeding mode and was associated with tract-wide differences in alpha and beta diversity across the duodenum, jejunum, ileum, and rectum. Because AF simultaneously involved fermented feed, separation from parents, loss of crop milk, altered feeding rhythm, and modified parental microbial transmission, the present design supports interpretation at the system-comparison level rather than attribution to fermentation alone.

At the compositional level, AF was associated with consistent segmental shifts in dominant lineages, most prominently reflected by redistribution between Firmicutes_D and Firmicutes_A and enrichment of *Lactobacillus* and *Limosilactobacillus*. Such taxa are commonly linked to fermentation-associated dietary environments and to epithelial and metabolic homeostasis in poultry and other monogastric species [14,15,16,17,18,30,31]. Nevertheless, the present observations remain associative, and the specific contribution of fermented substrate exposure versus altered feeding ecology cannot be separated in this design.

LEfSe consistently identified *Limosilactobacillus* as the most discriminant genus across all four segments, and exploratory network analysis placed *Lactobacillus* and *Limosilactobacillus* in central positions. Treatment-specific subnetwork summaries further indicated that AF-associated subnetworks contained more vertices and edges than PF subnetworks across segments but had lower density, suggesting a broader yet more dispersed pattern of microbial co-variation under the AF system. These findings are consistent with the possibility that lactobacilli-related taxa form an important component of the AF-associated microbial configuration [32,33,34]. However, because the co-occurrence networks were derived from correlation analyses in compositional 16S data, and because two PF subnetwork summaries were based on n = 5 rather than n = 6 available outputs, these patterns should be interpreted as descriptive topological summaries rather than evidence of direct ecological interaction.

Microbiota differences co-occurred with changes in intestinal morphology, particularly in the jejunum. AF was associated with greater villus height and a higher villus height-to-crypt depth ratio, suggesting altered mucosal development during the transition period. Such structural differences may reflect differences in nutrient presentation, luminal acidity, microbial metabolites, or feeding behavior under AF relative to PF [35,36].

Beyond intestinal structure, AF was associated with coordinated differences in muscle development and lipid-related phenotypes [37,38,39]. Histological analysis showed larger breast muscle fiber dimensions, and chemical analysis indicated higher intramuscular fat content together with higher eicosadienoic acid, eicosapentaenoic acid, α-linolenic acid, docosahexaenoic acid, and linoleic acid in breast muscle. These differences may be consistent with altered nutrient partitioning or lipid metabolism under the AF system.

AF also increased the organ indices of the heart, liver, lungs, and bursa of Fabricius and altered serum lipid-related indicators. The lower semi-eviscerated yield observed in AF may be related to this disproportionate increase in visceral organ indices, particularly the heart, liver, and lungs, which indicates that a larger fraction of body mass was allocated to internal organs. Because dressing percentage, full-eviscerated yield, breast muscle yield, thigh muscle yield, and abdominal fat percentage were not significantly changed, the lower semi-eviscerated yield is more likely to reflect altered tissue allocation under the AF system than impaired carcass or muscle development. The exploratory Spearman heatmap showed co-variation between lactobacilli-related genera and several of these traits. Because these correlations were based on numerous unadjusted pairwise tests and a limited number of replicate units, they should be regarded as hypothesis-generating rather than confirmatory. Similarly, PICRUSt2 suggested differences in predicted pathways related to carbohydrate, amino acid, lipid, and cofactor/vitamin metabolism, but such outputs represent inferred functional potential from 16S data rather than directly measured function.

Several limitations should be acknowledged. First, AF differed from PF in both diet fermentation and feeding mode (separation from parents, loss of crop milk, handling frequency, feeding schedule, and parental microbial transmission); therefore, the present design does not isolate the effect of fermentation alone. Second, the intervention lasted only 7 days, from 18 to 25 days of age, which corresponds to the late stage of the transition from crop milk to solid feed. This relatively short period was sufficient to detect short-term microbial and developmental associations but does not allow inference about long-term effects on growth performance, health, or later production traits. Third, the study used six replicate units per treatment, and some downstream measurements relied on pooled or representative samples at the replicate level, which limits statistical power and individual-level inference. Fourth, the representative jejunal histological images should be interpreted cautiously because some local villus disruption, particularly in the PF panel, may derive from tissue-processing artifact rather than biological treatment differences. For this reason, the quantitative morphology data were given greater interpretive weight than the appearance of any single representative field. Fifth, the correlation heatmap and co-occurrence networks were exploratory analyses of compositional 16S data and should not be interpreted causally. Sixth, PICRUSt2 provided only predicted functional potential, and the underlying feed organic acids and multi-omics readouts were not measured. Future studies using longer-term factorial feeding designs together with shotgun metagenomics and metabolomics are needed to validate mechanisms.

## 5. Conclusions

In conclusion, the AF system (artificial feeding with a lactic acid bacteria-fermented diet) differed from the PF system in feed physicochemical properties and feeding ecology and was associated with tract-wide differences in microbiota diversity and composition across the duodenum, jejunum, ileum, and rectum. AF relative to PF was also associated with greater jejunal villus height and VH/CD, larger muscle fiber dimensions, higher intramuscular fat, altered selected polyunsaturated fatty acids and serum lipid-related indicators, lower semi-eviscerated yield, and higher organ indices, including the bursa of Fabricius. *Limosilactobacillus* emerged as the most discriminant genus across segments, whereas *Lactobacillus* and *Limosilactobacillus* occupied central positions in exploratory network analyses; treatment-specific subnetwork metrics further suggested larger but less dense AF-associated microbial subnetworks. These findings define a reproducible AF-associated microbial and phenotypic profile in squabs, but they should not be interpreted as evidence that fermentation alone caused the observed changes or as evidence of long-term benefits beyond the 7-day intervention.

## Figures and Tables

**Table 1:** Ingredients and calculated/formulated nutrient composition of the basal diet (as-fed basis).

Ingredients	Content	Nutritional Level	Content
Corn, %	43.00	Metabolizable energy (ME, MJ/kg)	11.80
Soybean meal, %	30.00	Crude protein (CP, %)	≥17.0
Wheat, %	15.00	Crude ash (CA, %)	≤14.0
Sorghum, %	5.00	Crude fiber (%)	≤6.0
NaCl, %	0.30	Moisture (%)	≤12.9
Limestone (rock flour), %	3.00	Lysine (%)	≥1.5
Calcium hydrogen phosphate (CaHPO_4_), %	3.00	Phosphorus (P, %)	≥0.45
Soybean Oil, %	0.22	Sodium chloride (NaCl, %)	0.25–0.85
Premix, %	0.48	Calcium (Ca, %)	0.7–1.2
Total	100	Selenium (Se, mg/kg)	0.2–0.5

Note: The premix provides the following nutrients per kilogram of feed: Vitamin A 200 kIU; Vitamin D3 60 kIU; Vitamin E 700 IU; Vitamin K3 125 mg; Vitamin B1 38 mg; Vitamin B6 80 mg; Vitamin B12 0.4 mg; calcium pantothenate 50 mg; folic acid 0.75 mg; biotin 0.75 mg. Metabolizable energy (ME) is a calculated value; the remaining nutrient values are formulated/guaranteed values supplied by the manufacturer. Abbreviations: CP, crude protein; CA, crude ash; Ca, calcium; P, phosphorus; NaCl, sodium chloride; Se, selenium. Ingredient terms follow the manufacturer’s labeling; soybean refers to soybean meal.

**Table 2:** Changes in analyzed nutrient composition of the basal diet before fermentation and after fermentation (as-fed basis; dry-matter basis values are provided for key nutrients).

	Before Fermentation (As-Fed)	After Fermentation (As-Fed)
Crude protein (CP, %)	17.6	13.3
Moisture (%)	10.6	31
Crude ash (CA, %)	6.5	4.6
Crude protein (DM basis, %)	19.69	19.28
Crude ash (DM basis, %)	7.27	6.67
pH	6.3	4.4
Acid value (mg KOH/g)	3.6	27.8
Lactic acid bacteria (CFU/g)	ND	7 × 10^5^
Molds and yeasts (CFU/g)	ND	1 × 10^1^

Note: The pre-fermentation diet corresponds to the basal diet shown in Table 1. Values are presented as analyzed measurements. pH is unitless. Acid value is expressed as mg KOH/g. Microbial counts are expressed as CFU/g. Because fermentation increases moisture content, nutrient values should be compared on a dry-matter basis where applicable (see CP and CA on a dry-matter basis). Abbreviations: CP, crude protein; CA, crude ash; DM, dry matter; CFU, colony-forming units; ND, not detected.

**Table 3:** Effect of the AF system versus PF on slaughter performance of squabs.

Item	Treatments		*p*-Value
	PF	AF	
Dressing percentage	85.39 ± 0.68	86.86 ± 0.70	0.146
Semi-eviscerated yield	77.35 ± 0.76	72.88 ± 2.06	0.023
Full-eviscerated yield	62.66 ± 0.68	58.74 ± 1.88	0.065
Breast muscle yield	24.60 ± 0.71	24.39 ± 1.25	0.488
Thigh muscle yield	7.18 ± 0.18	7.41 ± 0.33	0.840
Abdominal fat percentage	1.29 ± 0.18	1.64 ± 0.18	0.182

Data are presented as mean ± SEM (n = 6).

**Table 4:** Effect of the AF system versus PF on meat quality of squabs.

Item	Treatments		*p*-Value
	PF	AF	
pH45 min	6.59 ± 0.07	6.89 ± 0.10	0.027
pH24 h	6.21 ± 0.07	6.36 ± 0.09	0.216
L*	41.21 ± 0.42	41.29 ± 0.41	0.900
a*	14.32 ± 0.32	14.77 ± 0.31	0.320
b*	9.38 ± 0.26	9.47 ± 0.27	0.811
Drip loss, %	3.03 ± 0.03	3.08 ± 0.04	0.331
Shear force, N	23.97 ± 1.31	25.58 ± 1.39	0.408

Data are presented as mean ± SEM (n = 6).

## Data Availability

Processed data supporting the findings of this study are available from the corresponding author on reasonable request. The raw reads generated by 16S rRNA amplicon sequencing have been deposited in the NCBI Sequence Read Archive (SRA) under BioProject accession number PRJNA1476791.

## Supplementary Materials

The following supporting information can be downloaded at https://www.mdpi.com/article/10.3390/ani16142145/s1, Figure S1: Rarefaction curves of the duodenal microbiota in squabs from the AF and PF systems; Figure S2: Rarefaction curves of the ileal microbiota in squabs from the AF and PF systems; Figure S3: Rarefaction curves of the jejunal microbiota in squabs from the AF and PF systems; Figure S4: rarefaction curves of the rectal microbiota in squabs from the AF and PF systems; Table S1: Differences between the AF and PF systems in body size traits of squabs; Table S2: Differences between the AF and PF systems in the nutritional composition of squab breast muscle; Table S3: Differences between the AF and PF systems in the fatty acid profile of squab breast muscle; Table S4: Differences between the AF and PF systems in organ indices of squabs; Table S5: Differences between the AF and PF systems in serum biochemical indicators of squabs; Table S6: Sequencing summary of all pooled intestinal-content samples from the duodenum, jejunum, ileum, and rectum; Table S7: LEfSe results for each intestinal segment comparing the AF and PF systems; Table S8: Segment-level and treatment-specific parameters and summary metrics of exploratory co-occurrence networks. Table S9: Segment-specific PERMANOVA results for Bray–Curtis beta diversity between the AF and PF systems in squabs.
