# Supplementary material for: The Artificial-Feeding System with a Lactic Acid Bacteria-Fermented Diet, Compared with Parent Feeding, Is Associated with Tract-Wide Microbiota Shifts and Coordinated Developmental Indices in Squabs
Source: Animals (Basel). 2026 Jul 10;16(14):2145. doi: 10.3390/ani16142145 (PMC13405684; doi:10.3390/ani16142145)
Supplement: Supplementary file 1 [file animals-16-02145-s001.zip › animals-4402566-supplementary.pdf]

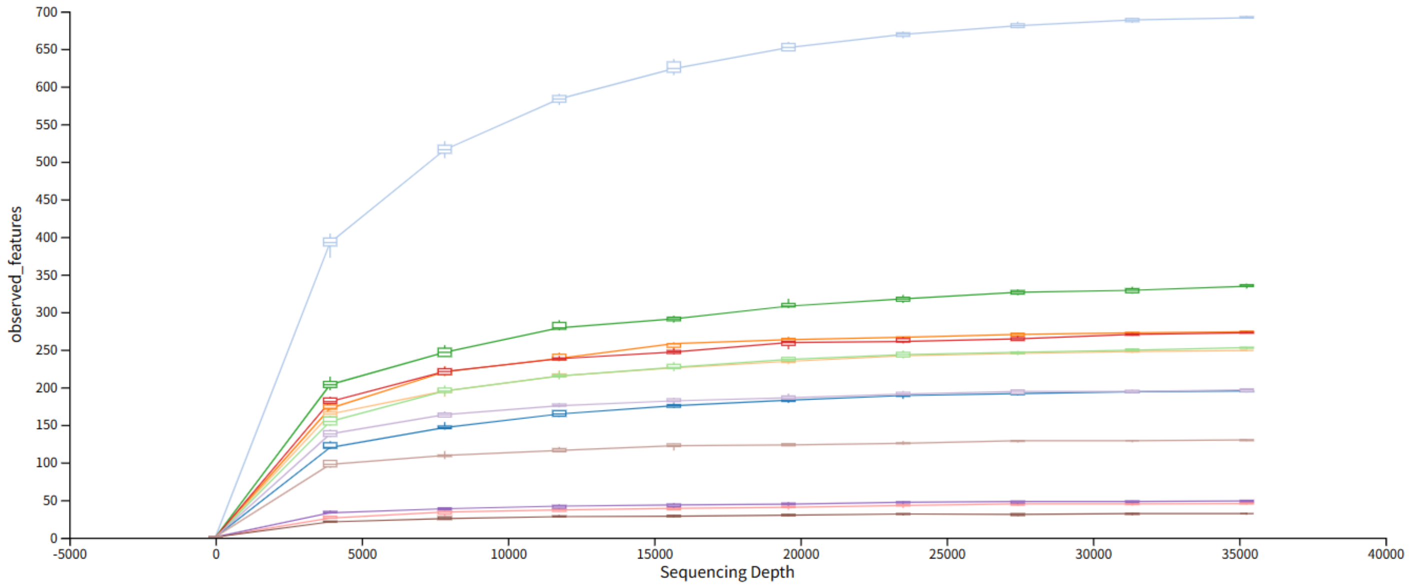

Figure S1. Rarefaction curves of the duodenal microbiota in squabs from the AF and PF systems. Curves were generated based on the ASV table after denoising and chimera removal. Each microbiota sample represents one pooled replicate. Curves approaching a plateau indicate that the sequencing depth was adequate to capture most of the bacterial diversity in the duodenal samples.

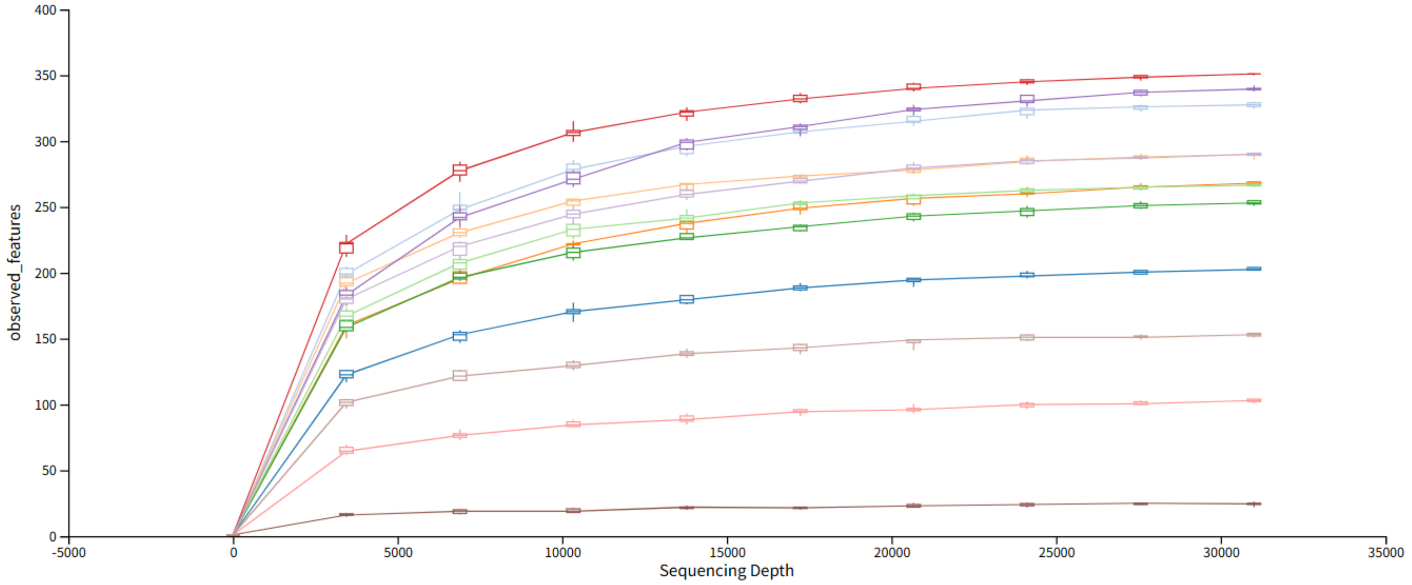

Figure S2. Rarefaction curves of the ileal microbiota in squabs from the AF and PF systems. Curves were generated based on the ASV table after denoising and chimera removal. Each microbiota sample represents one pooled replicate. Curves approaching a plateau indicate that the sequencing depth was adequate to capture most of the bacterial diversity in the ileal samples.

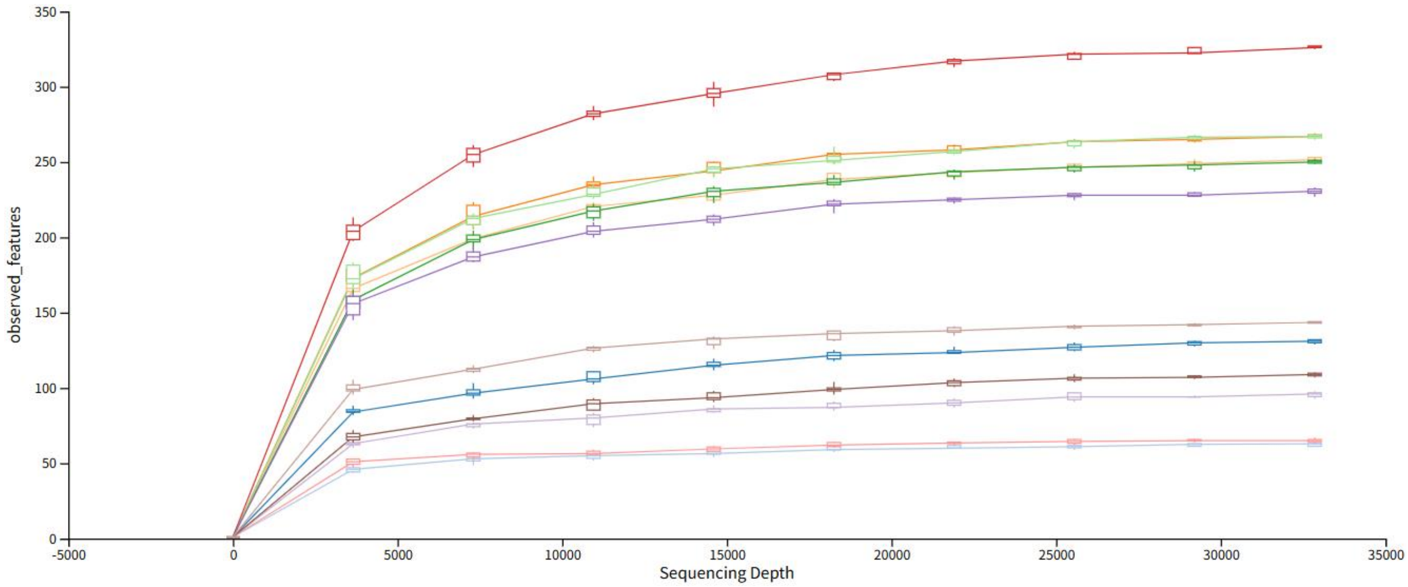

Figure S3. Rarefaction curves of the jejunal microbiota in squabs from the AF and PF systems. Curves were generated based on the ASV table after denoising and chimera removal. Each microbiota sample represents one pooled replicate. Curves approaching a plateau indicate that the sequencing depth was adequate to capture most of the bacterial diversity in the jejunal samples.

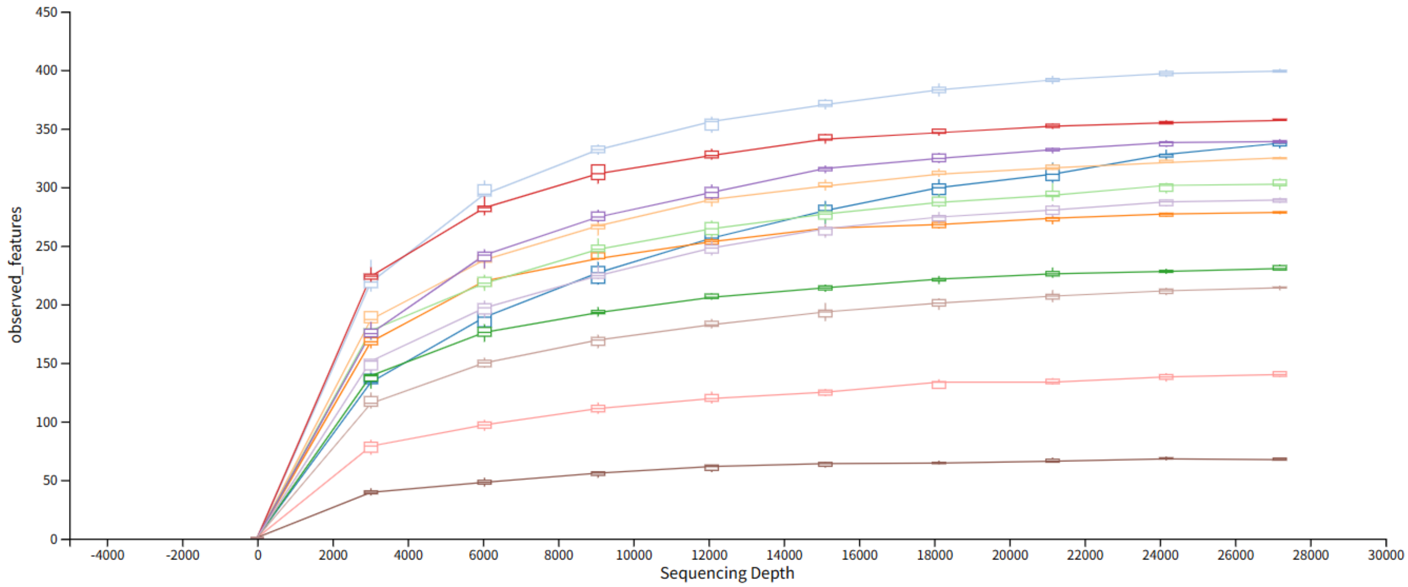

Figure S4. Rarefaction curves of the rectal microbiota in squabs from the AF and PF systems. Curves were generated based on the ASV table after denoising and chimera removal. Each microbiota sample represents one pooled replicate. Curves approaching a plateau indicate that the sequencing depth was adequate to capture most of the bacterial diversity in the rectal samples.

Table S1. Differences between the AF and PF systems in body size traits of squabs (cm).[1]

Item	Treatments		*P*-value
	PF	AF	
Body diagonal length	11.21 ± 0.10	11.31 ± 0.10	0.613
Keel length	8.39 ± 0.12	8.23 ± 0.10	0.243
Chest width	5.64 ± 0.07	5.85 ± 0.07	0.039
Chest depth	6.84 ± 0.08	7.27 ± 0.10	0.001
Pelvic width	4.53 ± 0.06	4.59 ± 0.08	0.597
Shank length	3.11 ± 0.04	3.16 ± 0.04	0.372
Shank circumference	2.30 ± 0.03	2.31 ± 0.03	0.735

Table S1 corresponds to Fig. 1.

[1] Values are presented as mean ± SEM (n = 6 replicates per treatment). PF, parent-feeding system; AF, artificial feeding with fermented feed system.

Table S2. Differences between the AF and PF systems in the nutritional composition of squab breast muscle (g/100 g fresh weight).[1]

Item	Treatments		*P*-value
	PF	AF	
Intramuscular fat	0.40 ± 0.00	0.70 ± 0.15	0.037
Met	0.53 ± 0.01	0.52 ± 0.02	0.507
His	0.59 ± 0.03	0.56 ± 0.01	0.261
Pro	0.60 ± 0.01	0.59 ± 0.03	0.507
Tyr	0.74 ± 0.02	0.74 ± 0.02	0.828
Ser	0.82 ± 0.02	0.81 ± 0.03	0.855
Phe	0.86 ± 0.02	0.84 ± 0.03	0.643
Gly	0.91 ± 0.02	0.89 ± 0.03	0.590
Thr	0.93 ± 0.03	0.92 ± 0.03	0.369
Ile	0.96 ± 0.03	0.95 ± 0.03	0.777
Val	0.97 ± 0.03	0.96 ± 0.03	0.816
Ala	1.19 ± 0.02	1.18 ± 0.04	0.791
Arg	1.38 ± 0.04	1.34 ± 0.04	0.268
Leu	1.72 ± 0.04	1.70 ± 0.06	0.369
Asp	1.85 ± 0.05	1.82 ± 0.06	0.736
Lys	2.18 ± 0.06	2.15 ± 0.07	0.813
Glu	2.95 ± 0.08	2.91 ± 0.09	0.753
Total amino acids	19.02 ± 0.50	18.90 ± 0.60	0.722
Protein	20.50 ± 0.42	20.43 ± 0.29	0.902
Moisture	74.10 ± 0.20	73.53 ± 0.17	0.099

Table S2 corresponds to Fig. 2A–E.

[1] Values are presented as mean ± SEM (n = 6 replicates per treatment). PF, parent-feeding system; AF, artificial feeding with fermented feed system. Met, methionine; His, histidine; Pro, proline; Tyr, tyrosine; Ser, serine; Phe, phenylalanine; Gly, glycine; Thr, threonine; Ile, isoleucine; Val, valine; Ala, alanine; Arg, arginine; Leu, leucine; Asp, aspartic acid; Lys, lysine; Glu, glutamic acid.

Table S3. Differences between the AF and PF systems in the fatty acid profile of squab breast muscle (g/100 g fresh weight).[1]

Item	Treatments		*P*-value
	PF	AF	
Eicosadienoic acid	0.0040 ± 0.0005	0.0066 ± 0.0003	0.008
Eicosapentaenoic acid	0.0050 ± 0.0002	0.0080 ± 0.0007	0.014
Gondoic acid	0.0055 ± 0.0005	0.0062 ± 0.0007	0.501
Myristic acid	0.0074 ± 0.0008	0.0084 ± 0.0007	0.426
Dihomo-γ-linolenic acid	0.0079 ± 0.0004	0.0071 ± 0.0004	0.266
α-linolenic acid	0.0104 ± 0.0011	0.0316 ± 0.0040	0.050
Docosahexaenoic acid	0.0135 ± 0.0015	0.0186 ± 0.0006	0.033
Arachidonic acid	0.1100 ± 0.0045	0.1277 ± 0.0055	0.069
Palmitoleic acid	0.1140 ± 0.0036	0.1115 ± 0.0085	0.906
Stearic acid	0.2140 ± 0.0080	0.2647 ± 0.0206	0.084
Palmitic acid	0.3897 ± 0.0237	0.4827 ± 0.0504	0.121
Linoleic acid	0.4157 ± 0.0253	0.7450 ± 0.0726	0.013
Oleic acid	0.5720 ± 0.0205	0.6740 ± 0.1048	0.827
Total fatty acids	1.8667 ± 0.0821	2.4900 ± 0.2793	0.099

Table S3 corresponds to Fig. 2F.

[1] Values are presented as mean ± SEM (n = 6 replicates per treatment). PF, parent-feeding system; AF, artificial feeding with fermented feed system.

Table S4. Differences between the AF and PF systems in organ indices of squabs (% of body weight).[1]

Item	Treatments		*P*-value
	PF	AF	
Heart	1.05 ± 0.03	1.40 ± 0.07	0.001
Liver	2.42 ± 0.06	3.28 ± 0.16	0.002
Spleen	0.14 ± 0.01	0.16 ± 0.04	0.617
Bursa of Fabricius	0.18 ± 0.01	0.24 ± 0.01	0.009
Pancreas	0.36 ± 0.01	0.34 ± 0.01	0.210
Lung	1.27 ± 0.02	1.44 ± 0.05	0.012

Table S4 corresponds to Fig. 3A.

[1] Organ indices were calculated as organ weight/body weight × 100. Values are presented as mean ± SEM (n = 6 replicates per treatment). PF, parent-feeding system; AF, artificial feeding with fermented feed system.

Table S5. Differences between the AF and PF systems in serum biochemical indicators of squabs.[1]

Item	Treatments		*P*-value
	PF	AF	
TP, g/L	22.00 ± 0.81	23.89 ± 1.20	0.214
ALT, U/L	18.63 ± 2.11	14.76 ± 1.43	0.074
TC, mmol/L	9.31 ± 0.66	9.14 ± 0.33	0.823
TG, mmol/L	1.09 ± 0.08	1.86 ± 0.22	0.002
HDL, mmol/L	4.11 ± 0.23	3.95 ± 0.11	0.518
LDL, mmol/L	1.10 ± 0.06	0.97 ± 0.07	0.208
ADP, mg/L	13.97 ± 0.66	8.92 ± 0.43	<0.001
LEP, ng/mL	4.39 ± 0.19	5.62 ± 0.38	0.011
TSH, μIU/mL	3.84 ± 0.36	4.78 ± 0.31	0.066
FAS, U/mL	20.32 ± 0.75	25.12 ± 1.67	0.026
HSL, U/mL	68.91 ± 3.93	100.26 ± 6.15	0.001
LPL, U/mL	45.20 ± 3.78	73.06 ± 3.50	<0.001

Table S5 corresponds to Fig. 3B.

[1] Values are presented as mean ± SEM (n = 6 replicates per treatment). PF, parent-feeding system; AF, artificial feeding with fermented feed system. TP, total protein; ALT, alanine aminotransferase; TC, total cholesterol; TG, triglyceride; HDL, high-density lipoprotein; LDL, low-density lipoprotein; ADP, adiponectin; LEP, leptin; TSH, thyroid-stimulating hormone; FAS, fatty acid synthase; HSL, hormone-sensitive lipase; LPL, lipoprotein lipase.

Table S6. Sequencing summary of all pooled intestinal-content samples from the duodenum, jejunum, ileum, and rectum.[1]

[1] Abbreviations: AF, artificial feeding with fermented feed system; PF, parent-feeding system. Each microbiota sample represented one pooled replicate. Raw reads indicate the number of input paired-end reads. Filtered reads indicate reads retained after quality filtering. Non-chimeric reads indicate reads retained after chimera removal. Final reads used for downstream analysis were based on the non-singleton ASV table. Across the 48 pooled samples, 3,311,262 raw reads were generated (55,220–90,873 reads/sample; mean 68,984.6). After singleton removal, 2,100,850 reads remained (28,622–61,263 reads/sample; mean 43,767.7).

SampleID	Group	Intestinal segment	Replicate ID	Input	Filtered	Denoised	Merged	Non-chimeric	Non-singleton	Retention rate after filtering (%)	Retention rate after chimera removal (%)	Final reads used for downstream analysis
PF_Duodenum_1	PF	Duodenum	1	78506	69692	69055	66497	38776	38765	88.77	58.31	38765
PF_Jejunum_1	PF	Jejunum	1	87727	79183	78937	77674	52834	52827	90.26	68.02	52827
PF_Ileum_1	PF	Ileum	1	80261	72484	72153	70845	41918	41912	90.31	59.17	41912
PF_Rectum_1	PF	Rectum	1	90873	82392	81736	78788	51359	51299	90.67	65.19	51299
PF_Duodenum_2	PF	Duodenum	2	76283	69338	69094	67867	54776	54773	90.9	80.71	54773
PF_Jejunum_2	PF	Jejunum	2	80891	73286	72908	72146	61214	61212	90.6	84.85	61212
PF_Ileum_2	PF	Ileum	2	77418	70511	70225	69693	60277	60277	91.08	86.49	60277
PF_Rectum_2	PF	Rectum	2	74173	67594	67317	65762	52028	52027	91.13	79.12	52027
PF_Duodenum_3	PF	Duodenum	3	76641	69050	68818	67927	60140	60139	90.1	88.54	60139
PF_Jejunum_3	PF	Jejunum	3	72748	66023	65757	64744	47148	47147	90.76	72.82	47147
PF_Ileum_3	PF	Ileum	3	74880	68037	67472	65244	38618	38595	90.86	59.19	38595
PF_Rectum_3	PF	Rectum	3	71068	64392	63673	59992	31725	31674	90.61	52.88	31674
PF_Duodenum_4	PF	Duodenum	4	79559	71542	71284	70082	53707	53707	89.92	76.63	53707
PF_Jejunum_4	PF	Jejunum	4	82377	74307	74204	73490	61263	61263	90.2	83.36	61263
PF_Ileum_4	PF	Ileum	4	70431	63378	63042	61614	42504	42496	89.99	68.98	42496
PF_Rectum_4	PF	Rectum	4	79019	71090	70400	67502	39390	39370	89.97	58.35	39370
PF_Duodenum_5	PF	Duodenum	5	69535	62724	62542	62238	60977	60976	90.2	97.97	60976
PF_Jejunum_5	PF	Jejunum	5	67294	58408	57878	55228	46302	46299	86.8	83.84	46299
PF_Ileum_5	PF	Ileum	5	63764	58109	57994	57755	55934	55934	91.13	96.85	55934
PF_Rectum_5	PF	Rectum	5	68863	62691	62372	61043	46263	46260	91.04	75.79	46260
PF_Duodenum_6	PF	Duodenum	6	66713	60345	59988	58763	46443	46434	90.45	79.03	46434
PF_Jejunum_6	PF	Jejunum	6	68880	62053	61837	60833	49385	49381	90.09	81.18	49381
PF_Ileum_6	PF	Ileum	6	63787	57551	57213	56629	45446	45440	90.22	80.25	45440
PF_Rectum_6	PF	Rectum	6	75341	68021	67596	65865	44744	44740	90.28	67.93	44740
AF_Duodenum_1	AF	Duodenum	1	70772	63443	62155	57006	37953	37901	89.64	66.58	37901
AF_Jejunum_1	AF	Jejunum	1	69552	55914	55705	54996	45692	45689	80.39	83.08	45689
AF_Ileum_1	AF	Ileum	1	67644	60587	60385	59527	42558	42549	89.57	71.49	42549
AF_Rectum_1	AF	Rectum	1	60336	53822	53346	51540	32774	32753	89.2	63.59	32753
AF_Duodenum_2	AF	Duodenum	2	61835	55404	55202	54962	37713	37708	89.6	68.62	37708
AF_Jejunum_2	AF	Jejunum	2	60768	54708	54506	54202	37131	37129	90.03	68.5	37129
AF_Ileum_2	AF	Ileum	2	60426	54454	54227	53687	36195	36186	90.12	67.42	36186
AF_Rectum_2	AF	Rectum	2	55220	49463	49132	48405	29637	29630	89.57	61.23	29630
AF_Duodenum_3	AF	Duodenum	3	58336	52483	52148	50218	37145	37117	89.97	73.97	37117
AF_Jejunum_3	AF	Jejunum	3	60031	54101	53788	53104	40816	40813	90.12	76.86	40813
AF_Ileum_3	AF	Ileum	3	60426	54439	54238	53916	37840	37832	90.09	70.18	37832
AF_Rectum_3	AF	Rectum	3	62381	56136	55502	53652	34410	34398	89.99	64.14	34398
AF_Duodenum_4	AF	Duodenum	4	73833	66066	65796	64917	49248	49241	89.48	75.86	49241
AF_Jejunum_4	AF	Jejunum	4	74811	67171	66929	66563	49861	49861	89.79	74.91	49861
AF_Ileum_4	AF	Ileum	4	58579	52841	52709	52349	37248	37241	90.2	71.15	37241
AF_Rectum_4	AF	Rectum	4	56879	50421	50332	50217	35683	35682	88.65	71.06	35682
AF_Duodenum_5	AF	Duodenum	5	59824	53601	53475	53222	39808	39806	89.6	74.8	39806
AF_Jejunum_5	AF	Jejunum	5	59177	53193	52971	52528	37447	37444	89.89	71.29	37444
AF_Ileum_5	AF	Ileum	5	59779	54051	53774	53197	34875	34873	90.42	65.56	34873
AF_Rectum_5	AF	Rectum	5	88238	81085	80785	79604	47462	47455	91.89	59.62	47455
AF_Duodenum_6	AF	Duodenum	6	63006	56388	56224	55625	40748	40747	89.5	73.25	40747
AF_Jejunum_6	AF	Jejunum	6	57904	52287	52040	51234	34585	34581	90.3	67.5	34581
AF_Ileum_6	AF	Ileum	6	58917	53012	52832	52146	32651	32645	89.98	62.61	32645
AF_Rectum_6	AF	Rectum	6	55556	49770	49519	48820	28631	28622	89.59	58.65	28622

Table S7. LEfSe results for each intestinal segment comparing the AF and PF systems.[1]

[1] Abbreviations: LEfSe, linear discriminant analysis effect size; AF, artificial feeding with fermented feed system; PF, parent-feeding system. Taxa with higher relative abundance in one group and meeting the LEfSe significance criteria are listed. Relative abundance values are presented as group means. LDA scores are shown on a log10 scale.

Intestinal segment	Taxonomic level	Taxon name	Full taxonomic annotation	Enriched group	Abundance (service output)	LDA score (log10)	P value
Duodenum	Genus	Limosilactobacillus	Bacteria.Firmicutes_D.Bacilli.Lactobacillales.Lactobacillaceae.Limosilactobacillus	AF	5.883328	5.537091	0.006392
Duodenum	Family	Lactobacillaceae	Bacteria.Firmicutes_D.Bacilli.Lactobacillales.Lactobacillaceae	AF	5.899058	5.399602	0.024975
Duodenum	Order	Lactobacillales	Bacteria.Firmicutes_D.Bacilli.Lactobacillales	AF	5.900291	5.397358	0.024975
Duodenum	Class	Bacilli	Bacteria.Firmicutes_D.Bacilli	AF	5.902755	5.39449	0.024975
Duodenum	Phylum	Firmicutes_D	Bacteria.Firmicutes_D	AF	5.902755	5.39449	0.024975
Duodenum	Family	Peptostreptococcaceae	Bacteria.Firmicutes_A.Clostridia.Peptostreptococcales.Peptostreptococcaceae	PF	5.431143	5.117933	0.014483
Duodenum	Order	Peptostreptococcales	Bacteria.Firmicutes_A.Clostridia.Peptostreptococcales	PF	5.431143	5.116331	0.015568
Duodenum	Genus	Romboutsia_B	Bacteria.Firmicutes_A.Clostridia.Peptostreptococcales.Peptostreptococcaceae.Romboutsia_B	PF	5.426156	5.112914	0.014483
Duodenum	Phylum	Bacteroidota	Bacteria.Bacteroidota	AF	4.284059	4.685071	0.003346
Duodenum	Class	Bacteroidia	Bacteria.Bacteroidota.Bacteroidia	AF	4.284059	4.67941	0.003346
Duodenum	Genus	Clostridium_T	Bacteria.Firmicutes_A.Clostridia.Clostridiales.Clostridiaceae.Clostridium_T	PF	3.309363	4.563244	0.020096
Duodenum	Genus	Dwaynesavagella	Bacteria.Firmicutes_A.Clostridia.Clostridiales.Clostridiaceae.Dwaynesavagella	AF	4.699239	4.545594	0.04951
Ileum	Genus	Limosilactobacillus	Bacteria.Firmicutes_D.Bacilli.Lactobacillales.Lactobacillaceae.Limosilactobacillus	AF	5.938553	5.52302	0.006485
Ileum	Family	Lactobacillaceae	Bacteria.Firmicutes_D.Bacilli.Lactobacillales.Lactobacillaceae	AF	5.947108	5.439575	0.010406
Ileum	Class	Clostridia	Bacteria.Firmicutes_A.Clostridia	PF	5.773669	5.380091	0.037373
Ileum	Phylum	Firmicutes_A	Bacteria.Firmicutes_A	PF	5.773669	5.380091	0.037373
Ileum	Family	Peptostreptococcaceae	Bacteria.Firmicutes_A.Clostridia.Peptostreptococcales.Peptostreptococcaceae	PF	5.573317	5.248492	0.002093
Ileum	Genus	Romboutsia_B	Bacteria.Firmicutes_A.Clostridia.Peptostreptococcales.Peptostreptococcaceae.Romboutsia_B	PF	5.568594	5.241404	0.002093
Ileum	Order	Peptostreptococcales	Bacteria.Firmicutes_A.Clostridia.Peptostreptococcales	PF	5.573317	5.23592	0.002093
Ileum	Genus	CCUG_7971	Bacteria.Firmicutes_A.Clostridia.Peptostreptococcales.Peptostreptococcaceae.CCUG_7971	PF	3.607346	5.169358	0.007397
Jejunum	Genus	Limosilactobacillus	Bacteria.Firmicutes_D.Bacilli.Lactobacillales.Lactobacillaceae.Limosilactobacillus	AF	5.93159	5.365485	0.024975
Jejunum	Family	Lactobacillaceae	Bacteria.Firmicutes_D.Bacilli.Lactobacillales.Lactobacillaceae	AF	5.986135	5.197097	0.006485
Jejunum	Order	Lactobacillales	Bacteria.Firmicutes_D.Bacilli.Lactobacillales	AF	5.986208	5.176337	0.006485
Jejunum	Class	Bacilli	Bacteria.Firmicutes_D.Bacilli	AF	5.986327	5.152758	0.010406
Jejunum	Phylum	Firmicutes_D	Bacteria.Firmicutes_D	AF	5.986327	5.152758	0.010406
Jejunum	Phylum	Fusobacteriota	Bacteria.Fusobacteriota	PF	2.305123	4.541859	0.02223
Jejunum	Order	Fusobacteriales	Bacteria.Fusobacteriota.Fusobacteriia.Fusobacteriales	PF	2.305123	4.520682	0.02223
Jejunum	Genus	Cetobacterium_A	Bacteria.Fusobacteriota.Fusobacteriia.Fusobacteriales.Fusobacteriaceae.Cetobacterium_A	PF	2.305123	4.518056	0.02223
Jejunum	Class	Fusobacteriia	Bacteria.Fusobacteriota.Fusobacteriia	PF	2.305123	4.517793	0.02223
Jejunum	Family	Fusobacteriaceae	Bacteria.Fusobacteriota.Fusobacteriia.Fusobacteriales.Fusobacteriaceae	PF	2.305123	4.516931	0.02223
Jejunum	Class	Campylobacteria	Bacteria.Campylobacterota.Campylobacteria	PF	2.459703	4.323891	0.02223
Jejunum	Genus	Helicobacter_E	Bacteria.Campylobacterota.Campylobacteria.Campylobacterales.Helicobacteraceae.Helicobacter_E	PF	2.459703	4.29572	0.02223
Jejunum	Family	Helicobacteraceae	Bacteria.Campylobacterota.Campylobacteria.Campylobacterales.Helicobacteraceae	PF	2.459703	4.28415	0.02223
Jejunum	Phylum	Campylobacterota	Bacteria.Campylobacterota	PF	2.459703	4.278509	0.02223
Jejunum	Order	Campylobacterales	Bacteria.Campylobacterota.Campylobacteria.Campylobacterales	PF	2.459703	4.253994	0.02223
Jejunum	Genus	Romboutsia_B	Bacteria.Firmicutes_A.Clostridia.Peptostreptococcales.Peptostreptococcaceae.Romboutsia_B	PF	4.293929	4.207302	0.007397
Jejunum	Order	Peptostreptococcales	Bacteria.Firmicutes_A.Clostridia.Peptostreptococcales	PF	4.306639	4.132706	0.007397
Jejunum	Family	Peptostreptococcaceae	Bacteria.Firmicutes_A.Clostridia.Peptostreptococcales.Peptostreptococcaceae	PF	4.306639	4.12833	0.007397

Rectum	Family	Lactobacillaceae	Bacteria.Firmicutes_D.Bacilli.Lactobacillales.Lactobacillaceae	AF	5.89589	5.586566	0.003948
Rectum	Family	Peptostreptococcaceae	Bacteria.Firmicutes_A.Clostridia.Peptostreptococcales.Peptostreptococcaceae	PF	5.899898	5.580792	0.002802
Rectum	Order	Peptostreptococcales	Bacteria.Firmicutes_A.Clostridia.Peptostreptococcales	PF	5.899898	5.57879	0.002802
Rectum	Genus	Limosilactobacillus	Bacteria.Firmicutes_D.Bacilli.Lactobacillales.Lactobacillaceae.Limosilactobacillus	AF	5.879452	5.577049	0.003948
Rectum	Genus	Romboutsia_B	Bacteria.Firmicutes_A.Clostridia.Peptostreptococcales.Peptostreptococcaceae.Romboutsia_B	PF	5.896898	5.576479	0.002802
Rectum	Class	Clostridia	Bacteria.Firmicutes_A.Clostridia	PF	5.942599	5.53266	0.003948
Rectum	Phylum	Firmicutes_A	Bacteria.Firmicutes_A	PF	5.942599	5.53266	0.003948
Rectum	Order	Lactobacillales	Bacteria.Firmicutes_D.Bacilli.Lactobacillales	AF	5.896048	5.526833	0.003948
Rectum	Class	Bacilli	Bacteria.Firmicutes_D.Bacilli	AF	5.89629	5.52463	0.003948
Rectum	Phylum	Firmicutes_D	Bacteria.Firmicutes_D	AF	5.89629	5.52463	0.003948
Rectum	Genus	CCUG_7971	Bacteria.Firmicutes_A.Clostridia.Peptostreptococcales.Peptostreptococcaceae.CCUG_7971	PF	3.730791	4.327469	0.004763
Rectum	Family	Turicibacteraceae	Bacteria.Firmicutes_D.Bacilli.Haloplasmatales_A.Turicibacteraceae	PF	3.514392	4.026965	0.002093
Rectum	Order	Haloplasmatales_A	Bacteria.Firmicutes_D.Bacilli.Haloplasmatales_A	PF	3.514392	4.026789	0.002093
Rectum	Genus	Turicibacter	Bacteria.Firmicutes_D.Bacilli.Haloplasmatales_A.Turicibacteraceae.Turicibacter	PF	3.514392	4.02615	0.002093

Table S8. Segment-level and treatment-specific parameters and summary metrics of exploratory co-occurrence networks constructed for each intestinal segment.

Part A. Segment-level empirical network metrics.

Intestinal segment	Feature level	Correlation algorithm	Filtering rule 1	Filtering rule 2	Threshold selection	Threshold value	Negative correlations removed for co-occurrence network	Community detection algorithm	Num_vertice	Num_edge	Average_nearest_neighbor_degree	Average_path_length	Degree_assortativity	Degree_centralization	Density	Cluster_num	Diameter	Transitivity	Modularity
Duodenum	ASV/OTU (service-provider network output)	SparCC	total sequence count ≥ 10	detected in ≥ 5 samples	random matrix theory	0.6	Yes	multi-level modularity optimization	263.0	4110.0	41.1088656191464	3.6765954278055	0.569082032025842	18343.0	0.119292949815691	9.0	10.3838287936927	0.836217391756761	0.565049347770437
Ileum	ASV/OTU (service-provider network output)	SparCC	total sequence count ≥ 10	detected in ≥ 5 samples	random matrix theory	0.6	Yes	multi-level modularity optimization	262.0	4101.0	40.9535739767411	3.67938558605926	0.571800959165052	18260.0	0.119943844871457	9.0	10.3838287936927	0.837855973187496	0.563791223664761
Jejunum	ASV/OTU (service-provider network output)	SparCC	total sequence count ≥ 10	detected in ≥ 5 samples	random matrix theory	0.6	Yes	multi-level modularity optimization	260.0	3954.0	39.9873446166432	3.66269125147117	0.596057011601682	17572.0	0.117433917433917	10.0	10.3838287936927	0.843460726016641	0.557306198812032
Rectum	ASV/OTU (service-provider network output)	SparCC	total sequence count ≥ 10	detected in ≥ 5 samples	random matrix theory	0.6	Yes	multi-level modularity optimization	262.0	4068.0	40.7454763695314	3.68178916137755	0.580598808551726	18064.0	0.118978678599631	9.0	10.3838287936927	0.840168236545656	0.562262101461416

Part B. Treatment-specific subnetwork summary metrics.

For PF Duodenum and PF Jejunum, one expected subnetwork file was absent from the uploaded service output; the means below are therefore based on the available n = 5 PF subnetworks for those segments.

Intestinal segment	Group	n subnetworks available	Samples included	Missing expected samples	Mean Num_vertice	Mean Num_edge	Mean Average_nearest_neighbor_degree	Mean Average_path_length	Mean Degree_assortativity	Mean Degree_centralization	Mean Density	Mean Cluster_num	Mean Diameter	Mean Transitivity	Mean Modularity
Duodenum	AF	6	AF_Duodenum_1, AF_Duodenum_2, AF_Duodenum_3, AF_Duodenum_4, AF_Duodenum_5, AF_Duodenum_6		164.667	1645.833	25.258	3.439	0.674583	6250.333	0.125608	9.667	8.101	0.832478	0.319492
Duodenum	PF	5	PF_Duodenum_1, PF_Duodenum_2, PF_Duodenum_3, PF_Duodenu	PF_Duodenum_5	51.0	292.4	13.483	1.944	0.113797	1095.6	0.304731	3.6	3.333	0.711977	0.30345

			m_4, PF_Duodenum_6												
Ileum	AF	6	AF_Ileum_1, AF_Ileum_2, AF_Ileum_3, AF_Ileum_4, AF_Ileum_5, AF_Ileum_6		184.167	1836.167	25.471	3.812	0.660196	7733.333	0.109885	8.333	8.888	0.813656	0.33797
Ileum	PF	6	PF_Ileum_1, PF_Ileum_2, PF_Ileum_3, PF_Ileum_4, PF_Ileum_5, PF_Ileum_6		66.333	413.0	13.486	2.134	0.150814	1885.5	0.178253	4.5	3.527	0.613294	0.394903
Jejunum	AF	6	AF_Jejunum_1, AF_Jejunum_2, AF_Jejunum_3, AF_Jejunum_4, AF_Jejunum_5, AF_Jejunum_6		149.833	1528.167	22.8	3.15	0.607308	5915.667	0.130522	7.167	6.947	0.816605	0.374225
Jejunum	PF	5	PF_Jejunum_1, PF_Jejunum_2, PF_Jejunum_3, PF_Jejunum_4, PF_Jejunum_6	PF_Jejunum_5	65.6	421.8	15.129	2.129	0.327504	1609.6	0.209863	4.0	4.084	0.727763	0.380261
Rectum	AF	6	AF_Rectum_1, AF_Rectum_2, AF_Rectum_3, AF_Rectum_4, AF_Rectum_5, AF_Rectum_6		181.5	1841.5	25.922	3.498	0.657551	7583.667	0.11258	9.0	7.982	0.814822	0.334695
Rectum	PF	6	PF_Rectum_1, PF_Rectum_2, PF_Rectum_3, PF_Rectum_4, PF_Rectum_5, PF_Rectum_6		54.0	323.667	13.258	2.05	0.006069	1253.333	0.24874	3.667	3.36	0.649114	0.349641

Table S9. Segment-specific PERMANOVA results for Bray-Curtis beta diversity between the AF and PF systems in squabs.

Intestinal segment	Sample size (n)	Permutations	pseudo-F	P-value	Q-value
ileum	12	999	4.123	0.002	0.002
jejunum	12	999	3.053	0.002	0.002
duodenum	12	999	3.805	0.002	0.002
rectum	12	999	21.368	0.002	0.002

Note: Values were obtained by PERMANOVA with 999 permutations based on Bray-Curtis dissimilarity matrices. The sample size represents the total number of pooled microbiota samples within each intestinal segment (n = 6 per treatment; total n = 12). P-values indicate AF-versus-PF differences within each intestinal segment, and Q-values are false discovery rate (FDR)-adjusted P-values across the four segment-level tests. AF, artificial feeding with fermented feed system; PF, parent-feeding system; pseudo-F, PERMANOVA pseudo-F statistic.
